# Differential impacts of *Cntnap2* heterozygosity and *Cntnap2* null homozygosity on axon and myelinated fiber development in mouse

**DOI:** 10.3389/fnins.2023.1100121

**Published:** 2023-01-30

**Authors:** Carmen Cifuentes-Diaz, Giorgia Canali, Marta Garcia, Mélanie Druart, Taylor Manett, Mythili Savariradjane, Camille Guillaume, Corentin Le Magueresse, Laurence Goutebroze

**Affiliations:** ^1^Inserm, Unité Mixte de Recherche (UMR)-S 1270, Paris, France; ^2^Faculté des Sciences et Ingénierie, Sorbonne University, Paris, France; ^3^Institut du Fer à Moulin, Paris, France

**Keywords:** Caspr2, corpus callosum, anterior commissure, sciatic nerve, axon diameter, myelin thickness, node of Ranvier, neuronal activity

## Abstract

Over the last decade, a large variety of alterations of the *Contactin Associated Protein 2 (CNTNAP2)* gene, encoding Caspr2, have been identified in several neuronal disorders, including neurodevelopmental disorders and peripheral neuropathies. Some of these alterations are homozygous but most are heterozygous, and one of the current challenges is to estimate to what extent they could affect the functions of Caspr2 and contribute to the development of these pathologies. Notably, it is not known whether the disruption of a single *CNTNAP2* allele could be sufficient to perturb the functions of Caspr2. To get insights into this issue, we questioned whether *Cntnap2* heterozygosity and *Cntnap2* null homozygosity in mice could both impact, either similarly or differentially, some specific functions of Caspr2 during development and in adulthood. We focused on yet poorly explored functions of Caspr2 in axon development and myelination, and performed a morphological study from embryonic day E17.5 to adulthood of two major brain interhemispheric myelinated tracts, the anterior commissure (AC) and the corpus callosum (CC), comparing wild-type (WT), *Cntnap2*^–/–^ and *Cntnap2*^+/–^ mice. We also looked for myelinated fiber abnormalities in the sciatic nerves of mutant mice. Our work revealed that Caspr2 controls the morphology of the CC and AC throughout development, axon diameter at early developmental stages, cortical neuron intrinsic excitability at the onset of myelination, and axon diameter and myelin thickness at later developmental stages. Changes in axon diameter, myelin thickness and node of Ranvier morphology were also detected in the sciatic nerves of the mutant mice. Importantly, most of the parameters analyzed were affected in *Cntnap2*^+/–^ mice, either specifically, more severely, or oppositely as compared to *Cntnap2*^–/–^ mice. In addition, *Cntnap2*^+/–^ mice, but not *Cntnap2*^–/–^ mice, showed motor/coordination deficits in the grid-walking test. Thus, our observations show that both *Cntnap2* heterozygosity and *Cntnap2* null homozygosity impact axon and central and peripheral myelinated fiber development, but in a differential manner. This is a first step indicating that *CNTNAP2* alterations could lead to a multiplicity of phenotypes in humans, and raising the need to evaluate the impact of *Cntnap2* heterozygosity on the other neurodevelopmental functions of Caspr2.

## Introduction

The *Contactin Associated Protein 2* gene (*CNTNAP2*), encoding the neuronal cell-adhesion transmembrane glycoprotein Caspr2, has gained prominence over the last decade in the field of neurological disabilities. A duplication of exon 4 of *CNTNAP2* was identified in two sisters suffering from Charcot–Marie–Tooth type 2, a peripheral motor and sensory neuropathy (Hoyer et al., 2015). Additionally, an increasing number of *CNTNAP2* alterations have been reported in patients with various neurodevelopmental disorders, including autism spectrum disorders (ASD), schizophrenia, intellectual disability, obsessive compulsive disorder, Pitt-Hopkins-like syndrome, attention deficit hyperactivity disorder, Gilles de la Tourette syndrome and cortical dysplasia focal epilepsy (Rodenas-Cuadrado et al., 2014; Poot, 2015, 2017; Saint-Martin et al., 2018). The genetic alterations identified in these disorders comprise a large diversity ranging from complex genomic rearrangements, homozygous and compound-heterozygous deletions and mutations, homozygous truncating non-sense mutations or deletions, and a large number of heterozygous missense variants found mainly in ASD patients. Currently, one of the major challenges is to estimate to what extent *CNTNAP2* alterations could affect the molecular and cellular functions of Caspr2, and therefore contribute to the development of the pathologies.

Caspr2 was initially identified as a component of the juxtaparanodal domains (juxtaparanodes) of the nodes of Ranvier in mature myelinated neurons, both in the central (CNS) and peripheral (PNS) nervous system (Poliak et al., 1999). Within the juxtaparanodes, Caspr2 is involved in the organization of specific axo-glial contacts, forming complexes with the cell-adhesion molecule Contactin2/TAG-1. These complexes are required for the clustering of the *Shaker*-type Kv1 (Kv1.1 and Kv1.2) channels (Poliak et al., 2003; Traka et al., 2003; Scott et al., 2017). Since Caspr2’s identification, several studies conducted in *Cntnap2*^–/–^ (KO) mice revealed that Caspr2 also plays critical roles in contributing to neuronal network formation during brain development, notably in neuronal migration (Penagarikano et al., 2011), dendritic arborization (Anderson et al., 2012; Gao et al., 2018), spine formation and maintenance (Gdalyahu et al., 2015; Varea et al., 2015; Lazaro et al., 2019), and synapse development and function (Anderson et al., 2012; Pinatel et al., 2015; Varea et al., 2015; Fernandes et al., 2019; Gao et al., 2019). Electrophysiological and imaging approaches further demonstrated that Caspr2 contributes to local and long-range brain connectivity (Penagarikano et al., 2011; Scott et al., 2017; Liska et al., 2018; Vogt et al., 2018; Zerbi et al., 2018; Antoine et al., 2019). Moreover, behavioral studies showed deficits in KO mice, some of which replicate clinical ASD phenotypes and are rescued by normalizing altered circuit connectivity (Penagarikano et al., 2011; Brunner et al., 2015; Scott et al., 2017; Choe et al., 2022). These data revealed mechanisms through which some *CNTNAP2* alterations may contribute to the development of neurological pathologies in humans.

However, studies in KO mice do not accurately replicate the genetic situations in patients and are not sufficient to fully explore the potential impact of the multiple *CNTNAP2* alterations identified thus far. Most of the genetic alterations are heterozygous and it is currently not known whether disruption of a single *CNTNAP2* allele could be sufficient to perturb the functions of Caspr2. Our recent observations indicate that some Caspr2 functions could be modulated by the level of the protein and affected by some ASD missense variants in a *Cntnap2*^+/–^ (HET) background (Canali et al., 2018). We showed that Caspr2 plays a dose-dependent function in cortical neuron axon growth *in vitro*, and that certain variants impinge Caspr2 cell adhesive properties and display loss of function, while others lead to protein retention in the endoplasmic reticulum and display a dominant-negative effect through oligomerization with wild-type (WT) Caspr2. Vogt et al. (2018) also found that HET fast-spiking PV^+^ cortical interneurons exhibit an intermediate electrophysiological phenotype between WT and KO interneurons. Nevertheless, whether or not the level of Caspr2 could more broadly modulate its functions remains to be determined.

In this study, we addressed this question, focusing on poorly explored functions of Caspr2 in axon development and myelination *in vivo*, whose disturbances could contribute to the development of both peripheral and central neurological disabilities. We performed a morphological study from embryonic day E17.5 to adulthood of two major brain interhemispheric myelinated tracts, the anterior commissure (AC) and the corpus callosum (CC), comparing WT, *Cntnap2* HET and KO mice. We also characterized myelinated fiber abnormalities in the sciatic nerves of mutant mice. Overall, our observations indicate that the level of Caspr2 modulates the development and organization of myelinated fibers both in the CNS and in the PNS.

## Materials and methods

### Animals

*Contactin Associated Protein 2 (Cntnap2)* mutant mice, previously described (Poliak et al., 2003), were obtained from the Jackson Laboratory and maintained in a C57BL/6J background. They were group-housed with *ad libitum* access to food and water and a 12–12 h light–dark cycle (light phase onset at 7 a.m.). For staging of embryos, the day of vaginal plug was considered E0.5.

### Tissue processing

For brain morphology analyses, E17.5 embryos were rapidly decapitated. Pups (P2, P7), juvenile (P30), and adult (P90) mice were deeply anesthetized by intraperitoneal injection of ketamine (100 mg/kg) and xylazine (10 mg/kg) and transcardially perfused with 4% paraformaldehyde (PFA) in 0.12 M phosphate buffer pH 7.4. Brains were fixed (embryos) or post-fixed (P2, P7, P30, P90) overnight (O/N) at 4°C in 4% PFA. Embryonic, P2 and P7 brains were washed in sodium phosphate buffered saline (PBS), embedded in 75 g/l gelatin and 100 g/l sucrose in PBS or in 15 g/l agarose and 20 g/l sucrose in PBS, and serial coronal (E17.5, P2, P7, 60 μm-thick) and sagittal (E17.5, 60 μm-thick; P7, 45 μm-thick) sections were obtained using a vibratome (Leica, France). Thirty-five μm-thick serial sagittal sections of P30 and P90 brains embedded in 35 g/l agarose and 80 g/l sucrose in PBS were obtained similarly. Forty μm-thick serial coronal sections of P30 and P90 brains and 500 μm-thick horizontal sections of P90 brains were performed without inclusion. All floating sections were conserved at 4°C in PBS with 1 g/l sodium azide until use.

For sciatic nerve immunostainings, 2-month-old mice were perfused as described above, sciatic nerves were dissected, fibers were teased apart on slides to yield single fiber preparations, air-dried and kept at −20°C.

### Immunostainings

For morphological analysis of the CC and AC, brain sections of E17.5, P2 and P7 animals were incubated in a permeabilization/saturation solution (PS1: PBS, 5 ml/l Triton X-100, 100 ml/l normal goat serum) for 1 h at room temperature (RT) and then O/N at 4°C (72 h for P7) with the anti-L1CAM antibody (Millipore, rat clone 324, #MAB5272, 1:400) diluted in PS1. Sections were then washed three times for 10 min in PBS/5 ml/l Triton X-100 (WS1) and incubated for 2 h at RT with adequate fluorophore-conjugated-secondary antibodies (Molecular Probes, Invitrogen, Waltham, MA, USA) diluted in PS1. For brain sections of P30 and P90 animals, permeabilization and saturation were performed in PBS supplemented with 2 g/l porcine skin gelatin and 2.5 ml/l Triton X-100 (PS2 solution), before incubation O/N at 4°C with the anti-NF-M antibody (Sigma, mouse clone NN18, #N5264, 1:300) diluted in PS2. The following day, sections were washed three times for 10 min in PBS/2.5 ml/l Triton X-100 (WS2) and incubated for 2 h at RT with secondary antibodies diluted in PS2. For all ages, after secondary antibody incubation, sections were washed three times for 10 min in WS1 (E17.5, P2, P7) or WS2 (P30, P90), and nuclei were stained for 10 min with Hoechst before mounting with Fluoromount-G medium (Invitrogen, Waltham, MA, USA). For neuron counting in the cortex, E17.5 sections were incubated with the anti-L1CAM antibody (1:750) together with the anti-Satb2 (Abcam, Cambridge, UK, mouse clone SATBA4B10, #ab51502, 1:750) and the anti-Ctip2 (Abcam, Cambridge, UK, rabbit #ab28448, 1:500) antibodies diluted in PS1.

Immunofluorescent staining of sciatic nerves was performed as described previously (Martin et al., 2017). Briefly, slides were treated with 0.1 M glycine for 30 min at RT or with methanol 50%/acetone 50% for 20 min at −20°C, pre-incubated for 1 h at room temperature in PS2, before incubation with primary antibodies diluted in PS2 overnight at 4°C (K_*v*_1.2 α subunit, mouse clone K67/25 #75-075, NeuroMab, 1:400; F3, goat #AF904, R&D Systems, 1:400; NrCAM, rabbit #ab24344, Abcam, Cambridge, UK, 1:300; TAG-1, goat #AF4439, R&D Systems, 1:500; Caspr (L51, 1:500) and Caspr2 (191, 1:400) previously described) (Menegoz et al., 1997; Denisenko-Nehrbass et al., 2003). After washing with PBS, coverslips were incubated for 2 h at room temperature with secondary antibodies diluted in PS2, washed again with PBS, and mounted with Fluoromount-G medium.

### Image acquisition and analysis

Images of immunostained brain sections or phase-contrast images were acquired using a macroscope MVX10 (Olympus, Tokyo, Japan). Thickness and area measurements were performed using the ImageJ software, for six mice/genotype/condition, except for AC area at E17.5 (four embryos/genotype). CC and cortex thicknesses were measured on one coronal section per brain per animal for E17.5 and P2 stages and three consecutive sections per animal for P90. Measurements were performed independently on both hemispheres and averaged; the measures were further averaged between the three consecutive sections for P90. Anterior branch (ACa) thickness at P90 was measured independently on both hemispheres on one horizontal section per brain per animal, and averaged. Measurements of CC and AC areas were performed on three consecutive sagittal sections per animal at all ages, and averaged.

For quantification of Satb2- or Ctip2-positive neurons at E17.5, images were acquired using a Leica SP5 confocal laser-scanning microscope (Leica microsystems, Watzlar, Germany, Z stack 1 μm, 25 stacks, 40× immersion objective) at the level of the somatosensory cortex of both hemispheres for two successive medial coronal sections per animal (*n* = 4 animals/genotype). The density of Satb2- or Ctip2-positive neurons was estimated using the Imaris software (Bitplane, Zurick, CH). To do so, images were cropped to exclude the region corresponding to the intermediate zone and the subplate layer, using L1CAM immunostaining to delineate these regions. The average volume of Satb2- and Ctip2-positive nuclei was calculated by averaging the volumes of 15 isolated nuclei. Masks were built to obtain the total volumes occupied by Satb2- and Ctip2-positive nuclei, respectively, excluding objects with a volume lower than the smallest volume out of the 15 used for averaging. The density of Satb2- or Ctip2-positive neurons was then calculated by dividing these total volumes by the average nucleus volume (Hoechst staining).

Images of stained sciatic nerve fibers were acquired using a Zeiss Celldiscoverer 7 imaging system for quantification analyzes, and a STELLARIS 5 confocal laser-scanning microscope (Leica Microsystems, Watzlar, Germany) to acquire representative images. Measurements of node length and node axonal diameter were performed manually on NrCAM stainings using the ImageJ software (3 mice/genotype, 81 nodes/mouse).

### Electron microscopy

For ultrastructural studies of the CC and the AC, P7 animals (3 pups/genotype) were deeply anesthetized by intraperitoneal injection of ketamine (100 mg/kg) and xylazine (10 mg/kg), and transcardially perfused with 4% PFA (EMS) and 2.5% glutaraldehyde (Microm Microtech, G003) in phosphate buffer 0.1 M pH 7.4. Brains were removed and post-fixed in the same fixative solution O/N at 4°C. P30 animals (3 mice/genotype) were anesthetized as above and transcardially perfused with 3% glutaraldehyde in Milloning’s phosphate buffer 0.1 M pH 7.4. Brains were removed and immediately processed. For the two ages, 1 mm-thick brain sagittal sections were produced and post-fixed for 1 h at 4°C in fresh fixative solution, washed once with phosphate buffer 0.1 M pH 7.4 (P7) or Milloning’s phosphate buffer 0.1 M (P30), and three times for 10 min with Palade buffer. Samples were then incubated in 2% osmium tetroxide in Palade buffer for 1 h at RT, rinsed in Palade buffer for 3 min and three times for 3 min in distilled water, dehydrated in a series of ethanol baths, and flat embedded in epoxy resin (EPON 812, Polysciences). After polymerization, 0.5 μm-thick semi-thin sections stained with toluidine blue to locate the CC and the AC. Then, blocks containing the CC and the AC were cut in 50 nm-thick ultrathin sections using an ultramicrotome (Ultracut E, Leica). Sections were examined with a Philips CM100 electron microscope and digital images acquired with a CCD camera (Gatan Orius). Diameters were calculated from areas measured manually using the Image J software (P7, ACa/ACp 300 axons/pup, CC 360 axons/pup; P30, ACa/ACp 147 myelinated fibers/mouse, CC 150 myelinated fibers/mouse). The percentage of myelinated fibers was determined by counting the numbers of unmyelinated and myelinated axons on 18 photos at magnification 9700×. The percentage of myelinated axons containing mitochondria was measured on six photos at magnification 2500×.

For ultrastructural analyses of peripheral myelinated fibers, the sciatic nerves were fixed *in situ* for ∼1 min with 3% glutaraldehyde in Milloning’s phosphate buffer 0.1 M pH 7.4, dissected out, placed in fixative O/N at 4°C, rinsed in PB, post-fixed in 2% osmium tetroxide in PB, dehydrated in an ascending series of ethanol, and embedded in epoxy resin. The global morphology of the nerves was evaluated on 0.5 μm-thick semi-thin transversal sections stained with toluidine blue and visualized with a DM6000 Leica microscope. Morphometric analyzes were performed on ultra-thin sections (50 nm) and examined as above (4 mice/genotype, 47 myelinated fibers/mouse).

### Brain lysate preparation and immunoblotting

To analyze protein levels, brains (six mice/genotype/age) were lysed in RIPA buffer containing proteases inhibitors and immunoblotting was performed as previously described (Canali et al., 2018). Briefly, equal amounts of proteins (40 μg) were loaded on NuPAGE 8–12% Bis-Tris gels (Thermo Fisher Scientific, Waltham, MA, USA) and transferred to 0.45 μm Nitrocellulose membranes. Membranes were blocked with 50 g/l non-fat dry milk in Tris-Buffered Saline solution/0.1 ml/l Tween 20 for 1 h at RT, incubated with primary antibodies in the same solution for 2 h at RT or O/N at 4°C, 1 h at RT with appropriate IRDye-conjugated secondary antibodies, and imaged and quantified using Odyssey Imaging System (LiCOR Biosciences, Lincolm, NE, USA). The commercial antibodies were from the following sources: anti-MBP, Serotec, rat clone 82–87, #MCA409S, 1:250; anti-PLP, Novus Biochemicals, rabbit, #NBP1-87781, 1:1000; anti-MAG, Zymed, rabbit, #34–6200, 1:1000; anti-GAPDH, Millipore, chicken, #AB2302, 1:5000. The anti-TAG-1 antibody has been described previously (Traka et al., 2003). The anti-Caspr2 was the same as the one used for immunostainings.

### RT-qPCR experiments

For mRNA expression analysis, total mRNA was extracted from brains (six mice/genotype/age) using the TRIzol™ Reagent (Invitrogen, Waltham, MA, USA) following the manufacturer’s recommendations. Reverse transcription was performed with the SuperScript II reverse transcriptase (Invitrogen, Waltham, MA, USA, #18064-022) and random primers, and qPCR in a Stratagene™ Mx3005P qPCR instrument (Agilent Technologies, Santa Clara, CA, USA) using the Brilliant II SYBR^®^ Green QPCR Master Mix (Agilent Technologies, Santa Clara, CA, USA, #600828). The MxPro QPCR Software (Agilent Technologies, Santa Clara, CA, USA) was used to perform expression analyses. The expression level of *Cntnap2* mRNA was normalized to *Psap* mRNA level. Primers for *Cntnap2* were designed on two 5′ exons: primer sense 5′CAGCGCTCTCGCTCTGGATT (accession number NM_001004357, nucleotides 261–280), primer anti-sense 5′CCCCAGCACCTCCTCGTTTATT (nucleotides 425–446), amplified DNA fragment 186 bp. Primers for *Psap* were the following: primer sense 5′CTGGTGTCAGAACATGGAGACTG (accession number NM_001146120, nucleotides 1708–1730), primer anti-sense 5′TGACTTCTGCAGCTGGGAAA (nucleotides 1781–1800), amplified DNA fragment 93 bp.

### Electrophysiology

#### Acute slice preparation

A total of 250 μm-thick coronal slices of the somatosensory cortex were prepared from brains of KO, HET, and WT littermates aged P10–P12. Acute coronal slices were cut using a vibroslicer (HM 650 V, Microm) in ice-cold artificial cerebrospinal fluid (ACSF) containing the following (in mM): 125 NaCl, 2.5 KCl, 25 glucose, 25 NaHCO3, 1.25 NaH2PO4, 2 CaCl2, and 1 MgCl2, continuously bubbled with 95% O_2_–5% CO_2_. Slices were incubated in ACSF at 32°C for 20 min and then at room temperature (20–25°C). For patch-clamp recordings, slices were transferred to the recording chamber where they were continuously superfused with ACSF (30–32°C).

#### Patch-clamp

Patch-clamp pipettes (4–6 Mohm resistance) were prepared from borosilicate glass (BF150-86-10; Harvard Apparatus, Holliston, MA, USA) using a DMZ pipette puller (Zeitz). Patch-clamp recordings were performed using an EPC-10 amplifier (HEKA Elecktronik GmbH, Reutlingen, Germany) with the following intracellular solution (in mM): 105 K-gluconate, 10 HEPES, 10 phosphocreatine-Na, 4 ATP-Na2, 30 KCl (pH 7.25, adjusted with KOH). Intrinsic excitability was measured in current-clamp using depolarizing current steps of increasing amplitude (0–500 pA, 500 ms) at an interstimulus interval of 6 s, from a cell potential set to −70 mV, in the presence of SR95531 hydrobromide (Gabazine, 10 μm, Hello Bio) to block GABA_*A*_ receptor-mediated synaptic transmission, 6-cyano-7-nitroquinoxaline-2,3-dione (CNQX, 10 μm, Biotrend, Köln, Germany) to block AMPA receptor-mediated synaptic transmission and D-2-amino-5-phosphonopentanoic acid (D-APV, 50 μm, Hello Bio) to block NMDA receptor-mediated synaptic transmission.

#### Data acquisition and analysis

Stimulus delivery and data acquisition were performed using the Patchmaster software (HEKA Elecktronik GmbH, Reutlingen, Germany). The junction potential (−5 mV) was left uncorrected. Signals were sampled at 20 kHz and filtered at 4 kHz. Offline analysis was performed using Igor Pro (WaveMetrics).

### Grid-walking test

Coordination between forelimbs and hind limbs and accurate limb placement were examined by assessing the ability to walk on metal grid bars with 1.5 cm gaps on the bottom of a 30 × 20 × 20 cm box (8–9 6-month-old males/genotype). The performance of each animal was analyzed by counting and averaging the number of errors in foot placement/total number of steps, during 2-min sessions, once a day, for three consecutive days. On the day before data collection, each mouse was allowed to walk on the grid for 2 min.

### Statistical analysis

Statistical analyses were performed with the GraphPad Prism 9 Software (GraphPad Software, San Diego, CA, USA). Results are provided as mean ± SEM or median ± quartile (Violin plot, medium smoothing). The normality of the samples was tested using the Shapiro–Wilk test. When the variables followed a normal distribution, statistical analyses were carried out using the unpaired *t*-test to compare 2 genotypes and the one-way ANOVA test to compare the three genotypes, followed by a Tukey’s multiple comparisons test. When the variables did not follow a normal distribution, the Mann–Whitney test was used to compare two genotypes and the Kruskal–Wallis test to compare the three genotypes, followed by a Dunn’s multiple comparisons test. The thicknesses of the CC measured on coronal section and the firings of cortical neurons were compared between genotypes using Two-way RM ANOVA tests, followed by a Tukey’s multiple comparisons test or a Sidak *post-hoc* test. Statistical analyses to compare the distributions of two quantitative variables were carried out using the Kolmogorov–Smirnov test. Linear regression analyzes were performed after checking the correlations between the variables (Pearson coefficient). The significance was established at a *P*-value < 0.05.

## Results

### Caspr2 level modulates CC morphology during development

**FIGURE 1:**
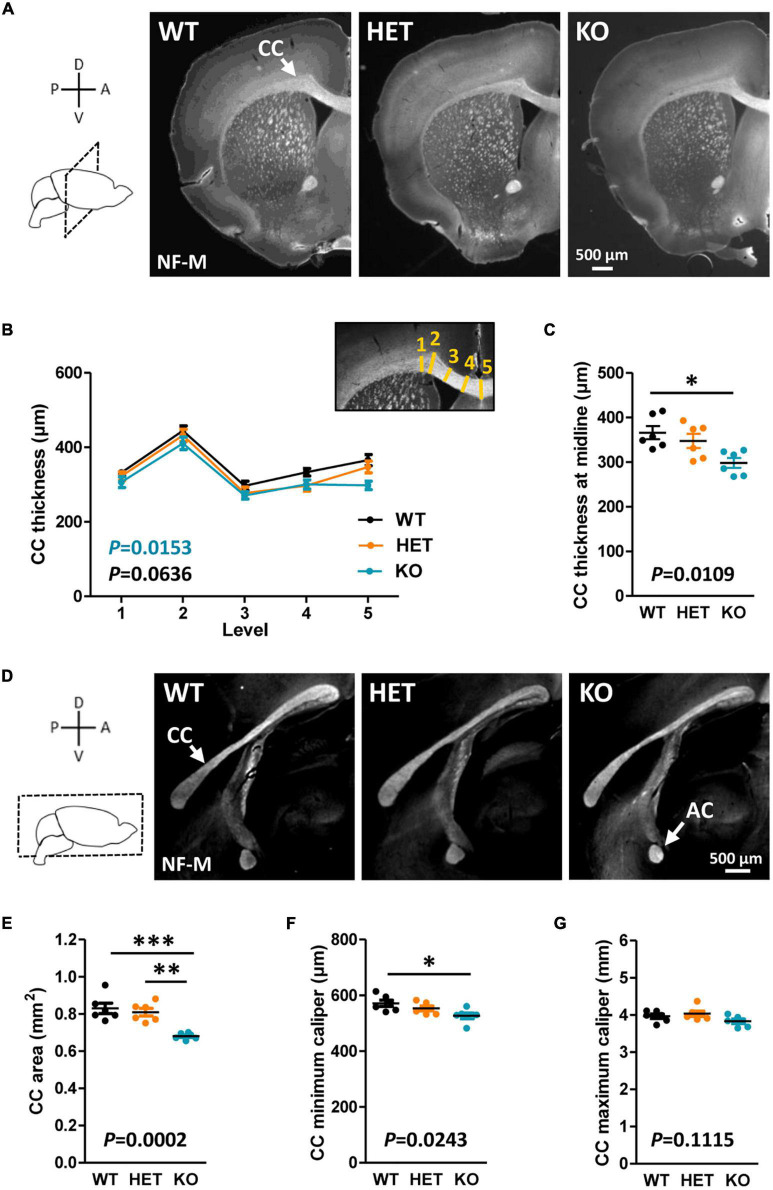
Corpus callosum (CC) morphology in adult mice. Representative images of brain coronal sections (Bregma + 0.50) of wild-type (WT), HET, and KO P90 mice showing CC immunostained with anti-NF-M antibodies **(A)**. CC thickness measured at different levels on coronal sections **(B)**; inset, schematic representation of the levels (yellow lines) where CC thickness measurements were performed. CC thickness at the midline of the brains **(C)**. Representative images of mid-sagittal brain sections of WT, HET, and KO P90 mice, showing the CC and the AC immunostained with anti-NF-M antibodies **(D)**. CC area **(E)**, minimum caliper **(F)**, and maximum caliper **(G)** measured on mid-sagittal brain sections. **(B,C,E–G)** Six animals/genotype, **(B)** average of measurements on both hemispheres on three consecutive sections/animal, **(C,E–G)** average of measurements on three consecutive sections/animal. Statistical tests: **(B)** Two-way RM ANOVA test; **(C,E–G)** Unpaired *t*-test to compare HET or KO mice to WT mice, and one-way ANOVA test to compare the three genotypes; *P < 0.05, ^**^P < 0.01, ^***^P < 0.001.

The CC is the most prominent white matter structure in the brain, formed by ∼80% of axons coming from neocortical neurons in layers II/III and ∼20% of axons coming from cortical neurons in layer V (Ku and Torii, 2020). A potential implication of Caspr2 in the development of this tract was suggested by our previous *in vitro* study showing a role for Caspr2 in axon growth of embryonic neocortical neurons (Canali et al., 2018). The morphology of the CC was first examined in WT, HET and KO mice at postnatal day P90 on brain coronal sections selected at Bregma +1.1, +0.55, and 0, and on mid-brain sagittal sections. Brain slices were immunostained with antibodies directed against the neurofilament subunit NF-M, which is expressed in commissural axons at late stages of development. From this point onward in the manuscript, the analyses were performed by comparing the three genotypes to investigate dose-dependent effects, but also by comparing HET and KO mice individually to WT mice to reveal potential effects of *Cntnap2* heterozygosity and/or null homozygosity, which would not be revealed by three-genotype comparisons. Main statistical results were reported on figures in black for three-genotype comparisons, and in color for individual comparisons to WT conditions when the difference was significant (HET, orange; KO, blue; all statistical analyses reported in Supplementary Table 1). When measuring the thickness of the CC in different positions from the brain midline to the lateral parts of coronal brain sections (Figure 1B, inset), we did not detect significant differences when comparing the three genotypes at the three Bregma levels, but a significant decrease in KO mice as compared to WT mice when comparing the two at Bregma + 0.50 (Figures 1A, B; data not shown for brain sections at Bregma +1.1 and 0). Decreased thickness was particularly pronounced at the midline of the brain (Figure 1C). Immunostainings of mid-sagittal sections consistently showed a decrease of CC area in KO mice as compared to WT and HET mice, as well as a decreased minimum caliper as compared to WT mice (Figures 1D–G). In HET mice, none of the morphometric parameters were significantly different from those in WT mice.

**FIGURE 2:**
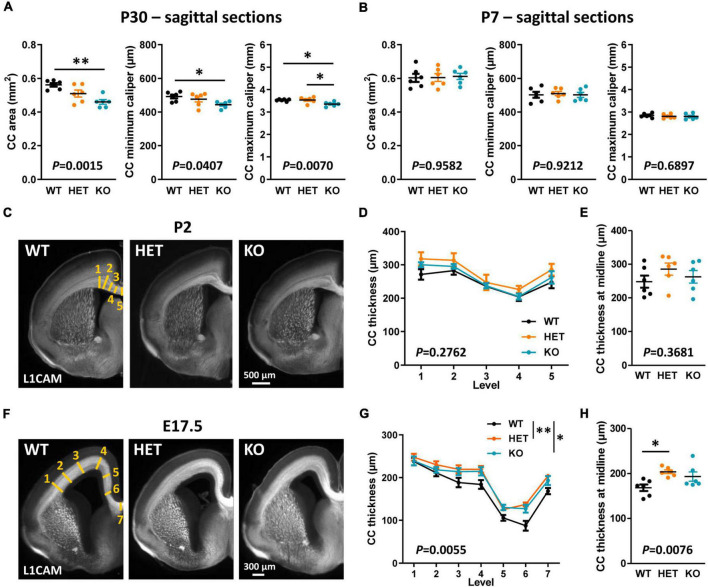
Corpus callosum (CC) morphology during development. CC area, minimum caliper, and maximum caliper measured on mid-sagittal brain sections of wild-type (WT), HET, and KO P30 mice **(A)** and P7 pups **(B)**. Representative images of brain coronal sections of WT, HET, and KO P2 pups **(C)** and E17.5 embryos **(F)** showing CC immunostained with anti-L1-CAM antibodies; the levels where CC thickness measurements were performed are schematized on the images of WT mice (yellow lines). CC thickness measured on coronal sections of P2 pups **(D)** and E17.5 embryos **(G)** at the levels indicated in panels **(C,F)**. CC thickness at the midline of the brains of P2 pups **(E)** and E17.5 embryos **(H)**. **(A,B,D,E,G,H)** Six animals/genotype, **(A,B)** average of measurements on three consecutive sections/animal, **(D,G)** average of measurements on both hemispheres on one section/animal, **(E,H)** measurements on one section/animal. Statistical tests: Unpaired *t*-test **(A,B,E)** or Mann–Whitney test **(H)** to compare HET or KO mice to WT mice, and one-way ANOVA test **(A,B,E)** or Kruskal–Wallis test **(H)** to compare the three genotypes; **(D,G)** Two-way RM ANOVA test followed by a Tukey’s multiple comparisons test **(G)**; *P < 0.05, ^**^P < 0.01.

Decreased CC thickness and area in adult KO mice were likely not due to a major decrease in the number of axons forming this tract since a previous study showed a normal distribution of the neocortical neurons projecting their axon through the CC in these mice (Scott et al., 2017). We attempted to find the temporal origin of the phenotype by characterizing the morphology of the CC through development. In mice, E15 pioneering axons from the cingulate cortex are the first axons forming the CC to reach the midline, followed later by neocortical projections at around E17.5 (Suarez et al., 2014). The formation of the CC continues after birth, until some axons begin to be myelinated by oligodendrocytes (OLs) (Suarez et al., 2014). The first myelinated sheaths are observable at P11 and a rapid phase of myelination occurs between P14 and P45 (Sturrock, 1980). We characterized CC morphology at P30 when myelination is ongoing, at P7 before the onset of myelination, at an early stage of axon development (E17.5), and at an intermediate developmental stage (P2). Morphometric analyzes were performed on mid-sagittal sections at P30 and P7, and on coronal sections at P2 and E17.5 because the structure of the CC at these stages did not allow measuring its area with confidence on sagittal sections. At P7, P2, and E17.5, brain sections were immunostained with antibodies directed against the cell-adhesion molecule L1CAM, which is widely expressed in commissural axons at embryonic and early post-natal developmental stages. The observations at P30 were comparable to those at P90, with a decrease in CC area and minimum and maximum calipers in KO mice as compared to WT (Figure 2A). In contrast, no difference was observed between the three genotypes either at P7 (Figure 2B) or at P2 (Figures 2C–E). Moreover, the thickness of the CC was remarkably increased in both HET and KO embryos at E17.5 as compared to WT embryos, with no significant difference between HET and KO embryos (Figures 2F, G). CC thickness at the brain midline was significantly increased in HET embryos as compared to WT embryos (Figure 2H). This increase was likely not due to an increased number of axons, since neither the thickness of the cortex nor the densities of callosal Ctip2^+^ and Satb2^+^ projecting neurons in the cortex were different between the three genotypes (Supplementary Figure 1). These observations demonstrate that the level of Caspr2 modulates the morphology of the CC during development, with a remarkable “switch” at P7, immediately before the onset of myelination. Furthermore, they strongly suggested that Caspr2 could play previously unidentified functions in axon development and/or myelination.

### Caspr2 level modulates AC morphology during development

**FIGURE 3:**
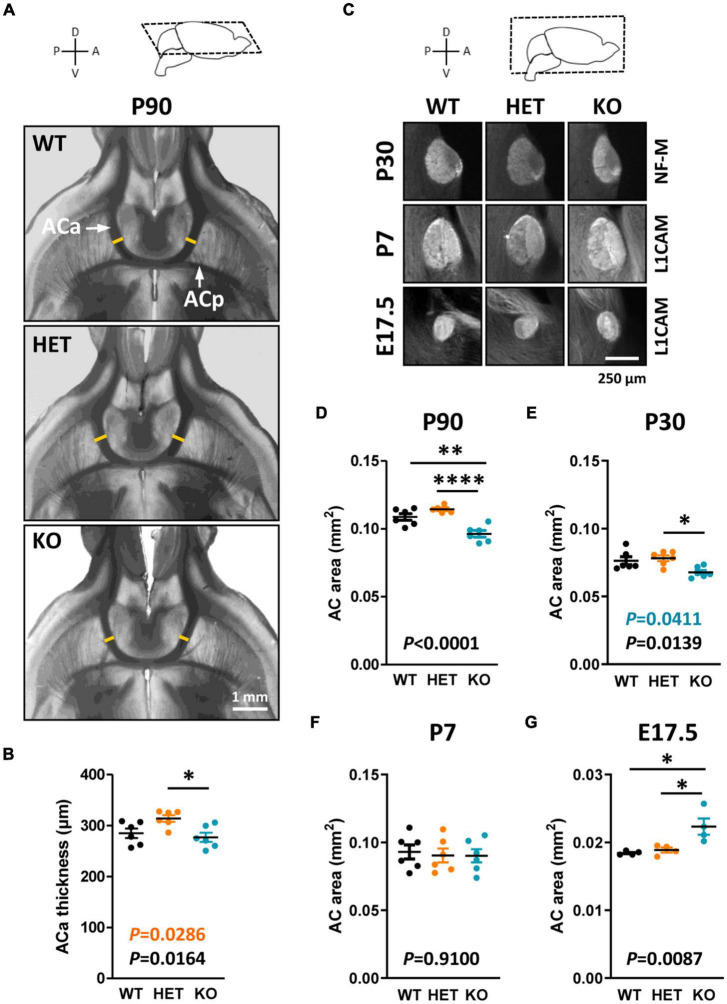
Anterior commissure (AC) morphology during development and at adulthood. Representative phase-contrast images of horizontal brain sections of wild-type (WT), HET, and KO P90 mice, showing the two branches of the AC **(A)**; yellow lines, levels where the thickness of the anterior branch (ACa) was measured. ACa thickness measured on horizontal brain sections of P90 mice **(B)**. Representative images of mid-sagittal brain sections of WT, HET, and KO mice at P30, P7, and E17.5, showing AC immunostained with anti-NF-M (P30) or L1CAM (P7, E17.5) antibodies **(C)**. AC area measured on mid-sagittal brain sections of P90 **(D)** and P30 **(E)** mice, P7 pups **(F)**, and E17.5 embryos **(G)**. **(B,D–F)** Six animals/genotype, **(G)** four animals/genotype, **(B)** average of measurements on both hemispheres on one section/animal, **(D–G)** average of measurements on three consecutive sections/animal. Statistical tests: Unpaired *t*-test **(B,D,F,G)** or Mann–Whitney test **(E)** to compare HET or KO mice to WT mice, and one-way ANOVA test **(B,D,F,G)** or Kruskal–Wallis test **(E)** to compare the three genotypes; *P < 0.05, ^**^P < 0.01, ^****^P < 0.0001.

The AC is formed by two main branches, contacting each other at the midline of the brain (Figure 3A). The ACa is composed mainly of axons from the anterior piriform cortex and the anterior olfactory nucleus, whereas the posterior branch (ACp) is composed mostly of axons from the posterior piriform cortex and the amygdala. We assumed that Caspr2 could contribute to the development and organization of this tract because *Cntnap2* expression was previously detected in the piriform cortex (Penagarikano et al., 2011; Gordon et al., 2016). As the AC follows a developmental timeline comparable to that of the CC in mice (Martin-Lopez et al., 2018), we characterized its morphology at the same developmental stages–P90, P30, P7, and E17.5. Phase-contrast images of 500 μm-thick horizontal brain sections did not reveal gross morphological defects in adult mutant mice (Figure 3A). However, we detected an increased thickness of the ACa in HET mice as compared to WT and KO mice, while the thickness tended to be decreased in KO mice, although not significantly different from that in WT mice (Figures 3A, B). This tendency turned out to be significant when considering the area of the whole AC on mid-sagittal sections (Figures 1D, 3D), suggesting that both the ACa and the ACp were thinner in KO mice as compared to WT mice. The area of the AC in KO mice appeared also significantly smaller than in HET mice. The AC area in HET mice was not significantly different from that in WT mice, but showed a strong tendency to increase (unpaired *t*-test, *P* = 0.0571), which was consistent with the increased thickness of the ACa. Results following a similar trend were observed in juvenile mice at P30 (Figures 3C, E). In contrast, no major differences were observed between the three genotypes in P7 pups (Figures 3C, F). In addition, the AC area was significantly increased in KO mice as compared to WT and HET mice in E17.5 embryos (Figures 3C, G). These data demonstrated that the level of Caspr2 also modulates the morphology of the AC during development, with a switch immediately before the onset of myelination as for the CC. They also suggested that Caspr2 could display functions in axon development and/or myelination extended to several myelinated tracts.

### Caspr2 level modulates axon diameter and myelin thickness of CC myelinated fibers in P30 mice

To further investigate the potential functions of Caspr2 in axon development and myelination, we first evaluated the timeline of overall myelination in HET and KO mice at four developmental stages from the onset of myelination (P10) to adulthood, by quantifying the levels of myelin proteins in whole brain extracts. Immunoblots showed a significant decrease of MBP, PLP, and MAG levels in KO mice at P30 as compared to WT mice, but not in P10, P15, and P90 mice (Supplementary Figures 2A–C), suggesting a potential widespread myelination delay in KO mice at P30. No alterations of myelin protein levels were detectable in HET mice, regardless of the developmental stage. We then performed tissue microdissection to compare myelin protein levels in the CC and the neocortex of P30 mice. Immunoblots revealed a significant decrease of MBP and MAG levels in the neocortex of KO mice as compared to WT mice (Supplementary Figures 2D, E), which was consistent with a delay of myelination previously reported in the neocortex of KO mice at P21 (Scott et al., 2017). However, no alteration of myelin protein level was detected in the CC of KO mice, in agreement with two previous studies showing similar MBP-immunoreactivity of the CC on brain coronal sections of WT and KO mice (Scott et al., 2017; Liska et al., 2018). No alteration of myelin protein level was detected in the CC of HET mice either.

**FIGURE 4:**
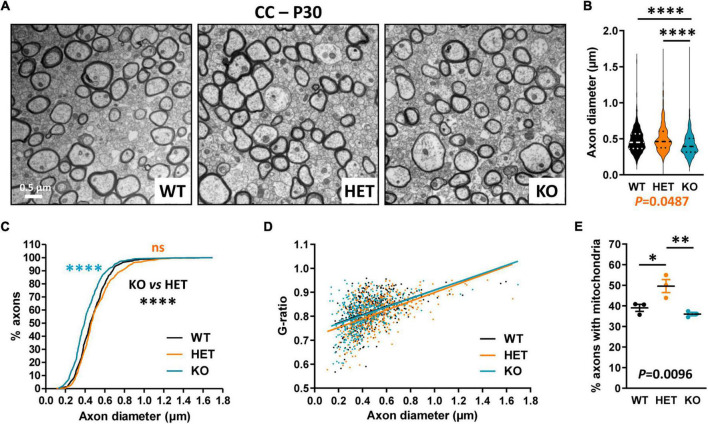
Ultrastructural abnormalities of corpus callosum (CC) myelinated fibers in P30 mice. Electron micrographs of transversal sections of the CC at brain midline, from wild-type (WT), HET, and KO P30 mice **(A)**. Axonal diameters of CC myelinated fibers **(B)**. Cumulative frequency distribution of axonal diameters (in %) **(C)**. Scatter plot graph displaying G-ratios of individual myelinated axons as a function of the respective axon diameters, and linear regression of the G-ratio measurements for each genotype **(D)**. Percentage of myelinated axons containing mitochondria **(E)**. **(B–D)** 450 myelinated fibers/genotype, 3 mice/genotype, 150 myelinated fibers/mouse; **(E)** 3 mice/genotype, % of mitochondria counted on ∼2000 axons/mouse. Statistical tests: **(B)** Mann–Whitney test to compare HET or KO mice to WT mice, and Kruskal–Wallis test to compare the three genotypes; **(C)** Kolmogorov–Smirnov test for pairwise comparisons; **(D)** Linear regression (WT *R*^2^ = 0.2102, HET *R*^2^ = 0.2871, KO *R*^2^ = 0.1959) for pairwise comparisons, HET vs. WT elevation *P* < 0.0001, HET vs. KO elevation *P* = 0.0005; **(E)** Unpaired *t*-test to compare HET or KO mice to WT mice, and one-way ANOVA test to compare the three genotypes; *P < 0.05, ^**^P < 0.01, ^****^P < 0.0001; ns, non-significant.

We further evaluated CC myelination in P30 mice at the ultrastructural level by measuring myelinated fiber and axon diameters, to subsequently calculate the G-ratio, which is the ratio of axon diameter to fiber diameter whose variations can reveal myelin thickness defects. Transversal sections were observed at ∼Bregma + 0.02, in dorsal projection of the AC. We detected a significant decrease in axon diameters in KO mice as compared both to WT and HET (Figures 4A–C), which was consistent with previous observations at Bregma −2.46 (Zerbi et al., 2018). No difference between KO and WT mice was detectable when plotting the G-ratios as a function of axon diameters, indicating absence of major myelination changes in KO mice (Figure 4D). No significant difference was observed either in the % of myelinated fibers between KO and WT mice (WT 16.5 ± 0.6775, KO 13.84 ± 2.594, unpaired *t*-test *P* = 0.3775, three mice/genotype), thus confirming that P30 KO mice do not present major myelination defects of the CC. In contrast, the % of myelinated fibers appeared significantly decreased in HET mice when compared to WT mice (WT 16.5 ± 0.6775, HET 8.747 ± 1.709, unpaired *t*-test *P* = 0.0135, three mice/genotype). In parallel, axon diameters were slightly decreased as compared to WT mice (Figures 4B, C). A significant difference was also detectable between WT and HET mice when plotting the G-ratios as a function of axon diameters (Figure 4D). The mean G-ratio was consistently decreased in HET mice as compared to WT mice, reflecting hypermyelination (WT 0.8200 ± 0.003069, HET 0.8099 ± 0.003189, Mann–Whitney test *P* = 0.0271, 450 fibers/genotype, three mice/genotype, 150 fibers/mouse). Interestingly, we also noticed a significant increase in the % of myelinated axons containing mitochondria in HET mice as compared to WT and KO mice (Figure 4E). Thus, our observations showed alterations of myelinated fibers in the CC of P30 HET mice which were not suspected by myelin protein level analyses, perhaps because the hypermyelination was masked by the decrease in the myelinated fiber number. Overall, axon diameter and myelin thickness modifications observed in mutant mice confirm the assumption that Caspr2 could play roles in axon development and/or myelination, which could be modulated by the protein level.

### Caspr2 level modulates axon diameter and myelin thickness of AC myelinated fibers in P30 mice

**FIGURE 5:**
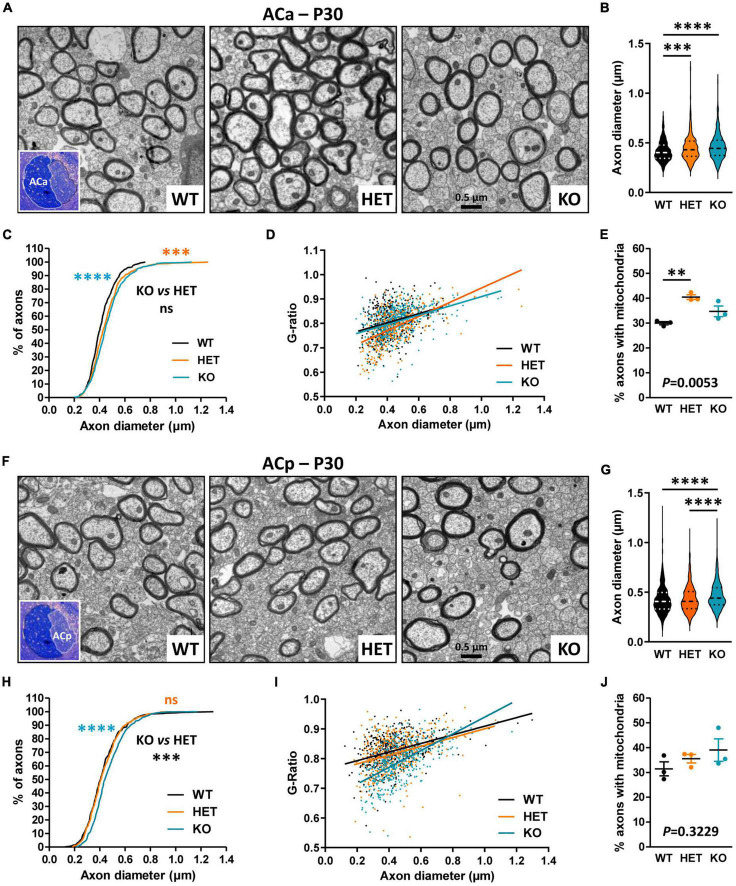
Ultrastructural abnormalities of anterior branch (ACa) and posterior branch (ACp) myelinated fibers in P30 mice. Electron micrographs of transversal sections of the ACa **(A)** and ACp **(F)** at brain midline, from wild-type (WT), HET, and KO P30 mice; insets, semi-thin transversal sections of the AC stained with toluidine blue, showing the ACa and the ACp. Axonal diameters of ACa **(B)** and ACp **(G)** myelinated fibers. Cumulative frequency distribution of axonal diameters (in %) of ACa **(C)** and ACp **(H)** myelinated fibers. Scatter plot graphs displaying G-ratios of individual myelinated axons as a function of the respective axon diameters, and the linear regression of the G-ratio measurements for each genotype, for the ACa **(D)** and the ACp **(I)**. Percentage of myelinated axons containing mitochondria in the ACa **(E)** and the ACp **(J)**. **(B–D,G–I)** 441 myelinated fibers/genotype, 3 mice/genotype, 147 myelinated fibers/mouse; **(E)** 3 mice/genotype, % of mitochondria counted on ∼2000 axons/mouse; **(J)** 3 mice/genotype, % of mitochondria counted on ∼1000 axons/mouse. Statistical tests: **(B,G)** Mann–Whitney test to compare HET or KO mice to WT mice, and Kruskal–Wallis test to compare the three genotypes; **(C,H)** Kolmogorov–Smirnov test for pairwise comparisons; **(D)** Linear regression (WT *R*^2^ = 0.08121, HET *R*^2^ = 0.3458, KO *R*^2^ = 0.1727) for pairwise comparisons, HET vs. WT slope *P* = 0.0024, HET vs. KO slope *P* = 0.0003; **(I)** Linear regression (WT *R*^2^ = 0.1589, HET *R*^2^ = 0.1062, KO *R*^2^ = 0.3420) for pairwise comparisons, HET vs. WT elevation *P* = 0.0047, KO vs. WT slope *P* < 0.0001, HET vs. KO slope *P* < 0.0001; **(E,J)** Unpaired *t*-test **(E)** or Mann–Whitney test **(J)** to compare HET or KO mice to WT mice, and one-way ANOVA test **(E)** or Kruskal–Wallis test **(J)** to compare the three genotypes; ^**^P < 0.01, ^***^P < 0.001, ^****^P < 0.0001; ns, non-significant.

We further evaluated whether Caspr2 could also display functions in axon development and/or myelination in the AC, searching similar axonal and/or myelination defects in mutant mice at P30. Ultrastructural analyses of the ACa and the ACp were performed separately, since the two branches are distinguishable on mid-brain transversal sections (Figures 5A, F, insets). No significant difference in the % of myelinated fibers was observed between the three genotypes, either in the ACa (WT 18.2 ± 1.554, HET 21.24 ± 0.611, KO 13.4 ± 3.022, one-way ANOVA test *P* = 0.0818, three mice/genotype) or in the ACp (WT 11.8 ± 1.02, HET 9.716 ± 0.7453, KO, 7.655 ± 0.8618, Kruskal–Wallis test *P* = 0.0857, three mice/genotype). In contrast, we detected modifications in axon diameters and myelination, which were remarkably different between the ACa and the ACp. While axon diameters of the ACa were significantly increased in both HET and KO mice as compared to WT mice, with an intermediate phenotype in HET mice (Figures 5B, C), axon diameters of the ACp were increased in KO mice only (Figures 5G, H). Additionally, plotting of the G-ratios as a function of axon diameters revealed a significant difference between HET mice and WT and KO mice for the ACa (Figure 5D), while both HET and KO mice were significantly different from WT mice for the ACp (Figure 5I). These observations were consistent with decreases of the mean G-ratios, demonstrating hypermyelination of ACa fibers in HET mice (WT 0.8060 ± 0.003146, HET 0.7854 ± 0.003246, KO 0.8077 ± 0.002936, Kruskal–Wallis test *P* < 0.0001, HET vs. WT *P* < 0.0001, HET vs. KO *P* < 0.0001, 441 fibers/genotype, 3 mice/genotype, 147 fibers/mouse), and of ACp fibers in both HET and KO mice, with an intermediate phenotype in HET mice (WT 0.8264 ± 0.005555, HET 0.8167 ± 0.002880, KO 0.7899 ± 0.003097, Kruskal–Wallis test *P* < 0.0001, KO vs. WT *P* < 0.0001, KO vs. HET *P* < 0.0001, HET vs. WT *P* = 0.0169, 441 fibers/genotype, 3 mice/genotype, 147 fibers/mouse). The % of myelinated axons of the ACa containing mitochondria was also significantly higher in HET mice than in WT (Figure 5E), while no difference was observed between the three genotypes for the ACp (Figure 5J). Altogether these data reinforce the assumption that Caspr2 could display functions in axon development and/or myelination of several myelinated tracts. In addition, they suggest that the underlying mechanisms may be different, depending on myelinated fiber type, and may therefore be differentially affected by variations in Caspr2 level.

### Caspr2 level modulates axon diameter and neuronal activity before and at the onset of myelination

**FIGURE 6:**
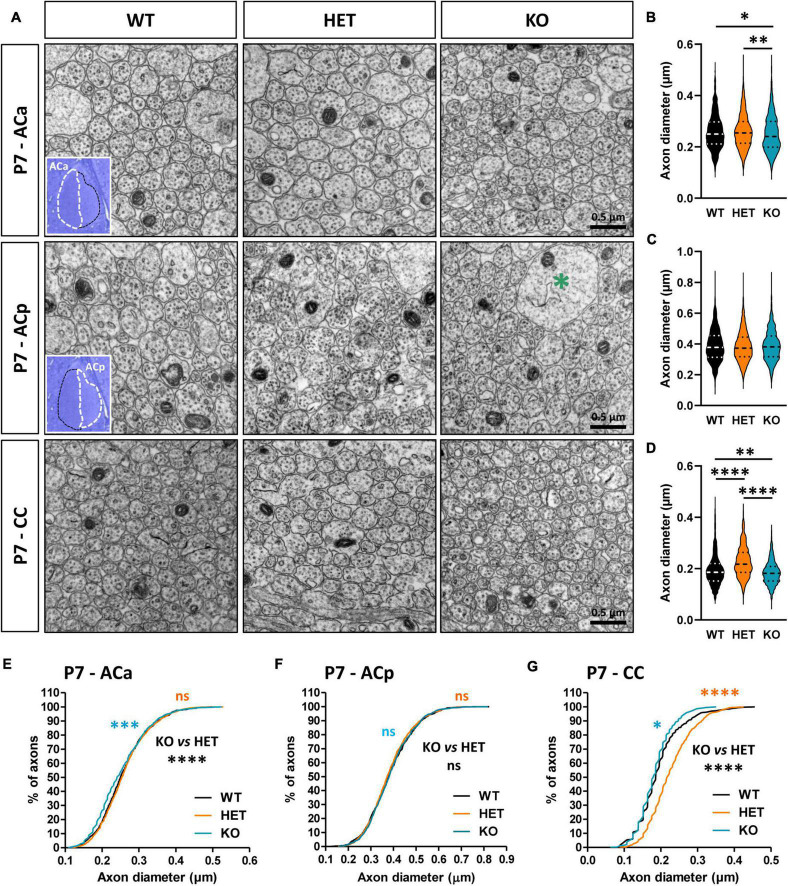
Anterior commissure (AC) and corpus callosum (CC) axon diameter abnormalities in P7 pups. Electron micrographs of transversal sections of the anterior branch (ACa), posterior branch (ACp), and CC at brain midline, from wild-type (WT), HET, and KO P7 pups; insets, semi-thin transversal sections of the AC stained with toluidine blue, showing the ACa and the ACp; the green asterisk indicates a growth cone **(A)**. Axonal diameters of the ACa **(B)**, ACp **(C)**, and CC **(D)**. Cumulative frequency distribution of axonal diameters (in %) of the ACa **(E)**, ACp **(F)**, and CC **(G)**. **(B,C,E,F)** 900 axons/genotype, 3 pups/genotype, 300 axons/pup; **(D,G)** 1080 axons/genotype, 3 pups/genotype, 360 axons/pup. Statistical tests: **(B–D)** Mann–Whitney test to compare HET or KO mice to WT mice, and Kruskal–Wallis test to compare the three genotypes; **(E–G)** Kolmogorov–Smirov test for pairwise comparisons; *P < 0.05, ^**^P < 0.01, ^***^P < 0.001, ^****^P < 0.0001; ns, non-significant.

Myelination in the CNS relies on a complex sequence of cellular events, which includes proliferation and migration of oligodendrocyte precursor cells (OPCs) in white matter tracts, recognition of target axons, and axon-glia signaling, differentiation of OPCs into mature myelinating OLs, axonal ensheathment, and myelin compaction. This sequence is controlled by intrinsic factors that are responsive to extracellular cues, including signaling molecules released by neurons or glial cells and cell-surface expressed proteins, and by neuronal activity, which regulates OPC proliferation and survival potentiating OL differentiation, and drives the extent of myelin formation (Stassart et al., 2018; de Faria et al., 2019). The changes in the morphology of the CC and AC in mutant mice at P7 suggested that Caspr2 could regulate some neuronal parameters immediately before and/or at the onset of myelination, which could control axo-glial cross-talk and, later, axon diameter and myelin thickness. To evaluate this hypothesis, we first compared the diameter of the axons between WT, HET, and KO pups at P7. Ultrastructural analyses did not reveal significant axon diameter difference between the three genotypes for the ACp (Figures 6A, C, F), but a slight decrease of axon diameters of the ACa in KO pups as compared to WT and HET pups (Figures 6A, B, E). In addition, axon diameters of the CC were significantly increased in HET pups as compared to WT and KO pups, and decreased in KO pups as compared to WT pups (Figures 6A, D, G).

**FIGURE 7:**
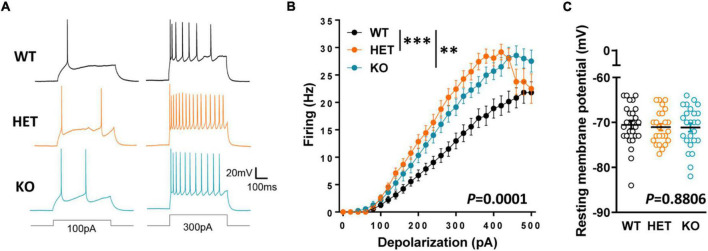
Intrinsic excitability in cortical pyramidal cells from P10–P12 pups. Sample spike trains evoked by a 100-pA (left) or a 300-pA somatic current injection (right) in a WT (top), HET (middle) and KO (bottom) neuron **(A)**. Mean f-i curve for WT (*n* = 27 cells from three mice), HET (*n* = 24 cells from three mice), and KO (*n* = 27 cells from three mice) **(B)**. Resting membrane potential of the recorded neurons **(C)**. Statistical tests: **(B)** Two-way RM ANOVA followed by Sidak’s *post-hoc* test; **(C)** One-way ANOVA to compare the three genotypes; ^**^P < 0.01, ^***^P < 0.001.

Following this observation, we explored whether Caspr2 could regulate the intrinsic excitability of layers III cortical neurons which project their axons in the CC at the onset of myelination, i.e., the propensity of neurons to fire action potentials when subjected to an input current. We measured the firing frequency of action potentials in response to 500 ms depolarizing current steps from a membrane potential of −70 mV, in the presence of blockers of synaptic activity. We found that intrinsic excitability was significantly increased in HET and KO neurons as compared to WT neurons (Figures 7A, B), while the resting membrane potential was unchanged (Figure 7C). The intrinsic excitability did not significantly differ between HET and KO mice. These results may, at least in part, reflect a role of Caspr2 at the axon initial segment, the site of initiation of action potentials, since Caspr2 has been detected in the axon initial segment of both pyramidal cells of human temporal neocortex (Inda et al., 2006) and rat hippocampal neurons in culture (Ogawa et al., 2008). Altogether our observations suggest that decreased Caspr2 dosage may indirectly control myelination through activity-dependent mechanisms, and by regulating axon diameter.

### Caspr2 levels are downregulated in HET mouse brain before the onset of myelination

**FIGURE 8:**
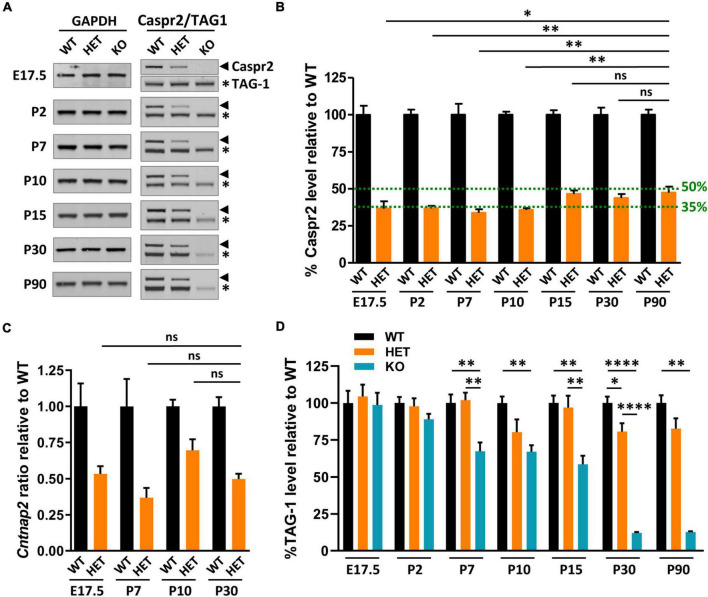
Caspr2, TAG-1, and *Cntnap2* mRNA levels in mouse brains during development. Representative immunoblots showing Caspr2, TAG-1, and GAPDH levels in brain lysates of wild-type (WT), HET, and KO mice at different developmental stages and at adulthood **(A)**. Levels of Caspr2 normalized to GAPDH levels and relative to mean WT (in %) **(B)**. Levels of *Cntnap2* mRNAs normalized to the *Psap* gene and relative to mean WT (ratio) in brain of WT, HET, and KO mice at E17.5, P7, P10, and P30 **(C)**. Levels of TAG-1 normalized to GAPDH levels and relative to mean WT (in %) **(D)**. **(B–D)** Six animals/genotype/age. Statistical tests: **(B)** Mann–Whitney test to compare Caspr2 levels in HET mice at stages E17.5, P2, P7, P10, P15, or P30, to Caspr2 level in HET mice at P90. **(C)** Unpaired *t*-test to compare *Cntnap2* mRNA levels in HET mice at stages E17.5, P7, or P10, to *Cntnap2* mRNA level in HET mice at P30. **(D)** One-way ANOVA test or Kruskal–Wallis test to compare the three genotypes at different ages; *P < 0.05, ^**^P < 0.01, ^****^P < 0.0001; ns, non-significant.

The impacts of *Cntnap2* heterozygosity on myelinated tract morphology, axon diameter, myelin thickness, and neuronal activity, either specific, opposite, or stronger than those of *Cntnap2* null homozygosity, led us to question Caspr2 levels in HET mice at different stages from E17.5 to adulthood. These levels were expected to be ∼50% relative to levels in WT brains. Immunoblots on whole brain extracts showed that this was the case at P15, P30, and P90, but not earlier during development (Figures 8A, B). Caspr2 levels were indeed reduced to ∼35% at E17.5, P2, P7, and P10, and significantly different from those in P90 HET mice. RT-qPCR experiments showed that this reduction may not be due to a decrease in gene expression (Figure 8C). *Cntnap2* mRNA level at P10 was actually increased and higher than that at P30 (unpaired *t*-test, *P* = 0.0564, six mice/genotype), indicating a need for cells to increase the level of Caspr2 around this developmental stage.

Decreased levels of cell-surface expressed proteins can be due to increased turnover and degradation, reflecting potential modifications in their interactions with their partners. We investigated this possibility, postulating that TAG-1 could be a partner of Caspr2 not only at juxtaparanodes of mature myelinated axons but also throughout development. Immunoblots on whole brain extracts of KO mice supported this assumption, showing that TAG-1 levels in these mice started to decrease from P2, to reach ∼33–42% decrease from P7 to P15, and ∼90% decrease at P30 and P90, as compared to WT mice (Figures 8A, D). In contrast, TAG-1 levels were not significantly affected in HET mice, except at P30. This indicates that the association of Caspr2 and TAG-1 are mostly preserved in HET mice and that the mechanisms which regulate Caspr2 levels at early developmental stages are probably independent of TAG-1. However, these data also suggest that the differential phenotypes observed in HET and KO could be partly due to the fact that the functions of TAG-1 are preserved in HET mice while they are highly perturbed in KO mice.

### Caspr2 level also modulates the organization of myelinated fibers in peripheral nerves

**FIGURE 9:**
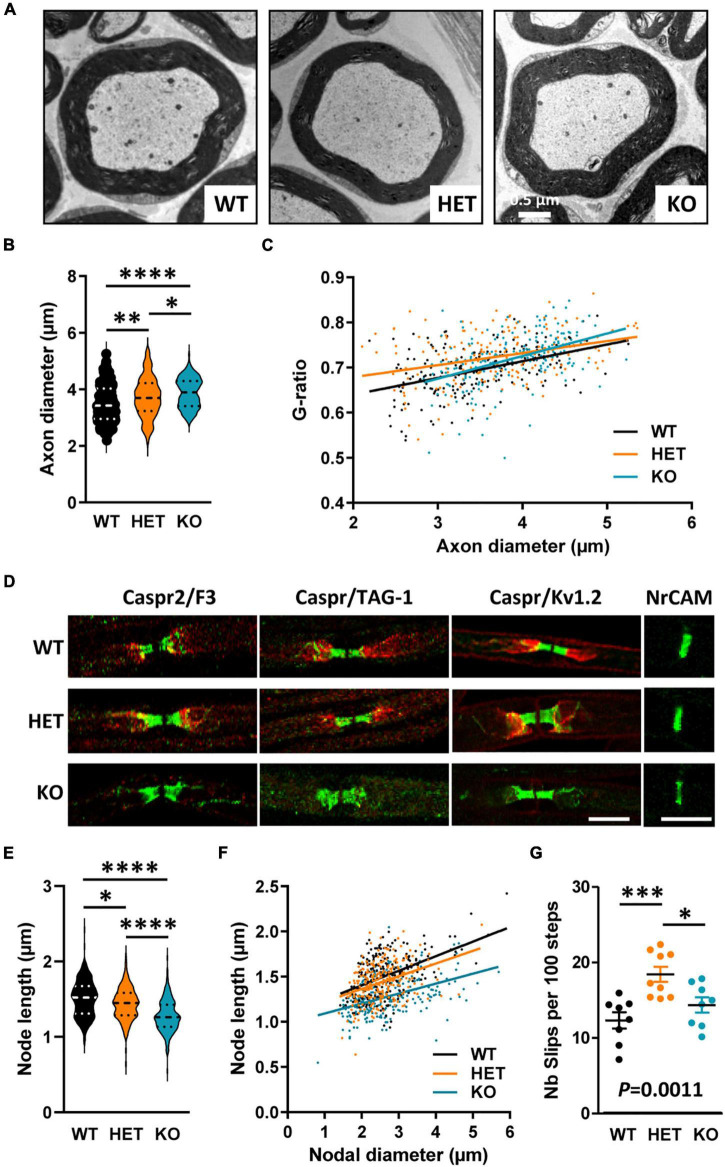
Myelinated fiber abnormalities in sciatic nerves of adult mice. Electron micrographs of transversal sections of the sciatic nerve of wild-type (WT), HET, and KO adult mice, showing a single myelinated fiber for each genotype **(A)**. Axonal diameters of myelinated fibers **(B)**. Scatter plot graph displaying G-ratios of individual myelinated axons as a function of the respective axon diameters, and the linear regression of the G-ratio measurements for each genotype **(C)**. Representative confocal images of immunostainings of sciatic nerves fibers from WT, HET, and KO adult mice, for nodal (NrCAM), paranodal (Caspr, F3), and juxtaparanodal (Caspr2, TAG-1, Kv1.2) proteins **(D)**. Length of the nodes measured on NrCAM immunostainings **(E)**. Scatter plot graph displaying length of individual node as a function of the respective nodal diameters, and the linear regression of the node length measurements for each genotype **(F)**. Number of slips per 100 steps made by WT, HET, and KO adult males during the grid-walking test **(G)**. **(B,C)** 141 myelinated fibers/genotype, 4 mice/genotype, 47 myelinated fibers/mouse, **(E,F)** 243 nodes/genotype, 3 mice/genotype, 81 nodes/mouse, **(G)** 8–9 mice/genotype. Statistical tests: **(B,E)** Kruskal–Wallis test to compare the three genotypes; **(C)** Linear regression (WT *R*^2^ = 0.2093, HET *R*^2^ = 0.1143, KO *R*^2^ = 0.2022) for pairwise comparisons, HET vs. WT elevation *P* < 0.0001, HET vs. KO slope *P* = 0.019; **(F)** Linear regression (WT *R*^2^ = 0.2295, HET *R*^2^ = 0.1368, KO *R*^2^ = 0.1935) for pairwise comparisons, HET vs. WT elevation *P* = 0.0052, KO vs. WT slope *P* = 0.0315, HET vs. KO elevation *P* < 0.0001; **(G)** one-way ANOVA test to compare the three genotypes; *P < 0.05, ^**^P < 0.01, ^***^P < 0.001, ^****^P < 0.0001. Bar scales, 10 μm **(E)**.

The identification of a *CNTNAP2* genetic alteration in Charcot–Marie–Tooth patients led us to finally question whether *Cntnap2* heterozygosity and null homozygosity could also perturb the organization of peripheral myelinated fibers in adult mice. Semi-thin transversal sections of sciatic nerves stained with toluidine blue did not reveal any major difference in the global morphology of the nerves between adult WT, HET, and KO mice, nor in the total number of axons (data not shown). The structure of the myelin appeared normal both in HET and KO mice (Figure 9A). A previous study reported comparable values of axon diameters and G-ratios in the sciatic nerves of WT and KO adult mice (Poliak et al., 2003). We re-evaluated these data by focusing our ultrastructural analysis on large caliber motor fibers only. We found that the axonal diameters were increased in HET and KO mice as compared to WT mice, with an intermediate phenotype in HET mice (Figure 9B). When plotting the G-ratios as a function of axon diameter, we did not detect any difference between KO and WT mice, but a significant difference between HET and WT mice (Figure 9C). The mean G-ratio was consistently increased in HET mice as compared to WT mice, reflecting hypomyelination (WT 0.6948 ± 0.003826, HET 0.7244 ± 0.004060, Mann–Whitney test *P* < 0.0001, 141 fibers/genotype, 4 mice/genotype, 47 fibers/mouse). Furthermore, fluorescent immunolabelings on teased myelinated fibers confirmed a drastic reduction of TAG-1 and Kv1.2 channel enrichment in the juxtaparanodes of KO mice (Figure 9D), as previously described (Poliak et al., 2003), but did not reveal obvious modification in the enrichment of Caspr2, TAG-1, and Kv1.2 in HET mice (proteins detected in more than 90% of the juxtaparanodes). The levels of TAG-1 and Kv1.2 in nerve extracts were not affected either in HET mice as compared to WT mice (data not shown). However, a morphometric analysis showed an unexpected decrease in the length of the nodes (labeled for NrCAM) in both HET and KO mice, with an intermediate phenotype for HET mice (Figures 9E, F), while no difference was observed for the axonal diameters of the nodes (WT 2.640 ± 0.045 μm, HET 2.535 ± 0.036 μm, KO 2.677 ± 0.054 μm, Kruskal–Wallis test *P* = 0.5342, three mice/genotype). We questioned whether defects in sciatic nerve organization could lead to motor and coordination deficits in a grid-walking test. Interestingly, adult HET mice made significantly more slips than WT and KO mice during the 2 min of the test (Figure 9G). Altogether these observations demonstrate that Caspr2 level also modulates the organization of mature peripheral myelinated fibers, with potential functional consequences in HET mice.

## Discussion

**FIGURE 10:**
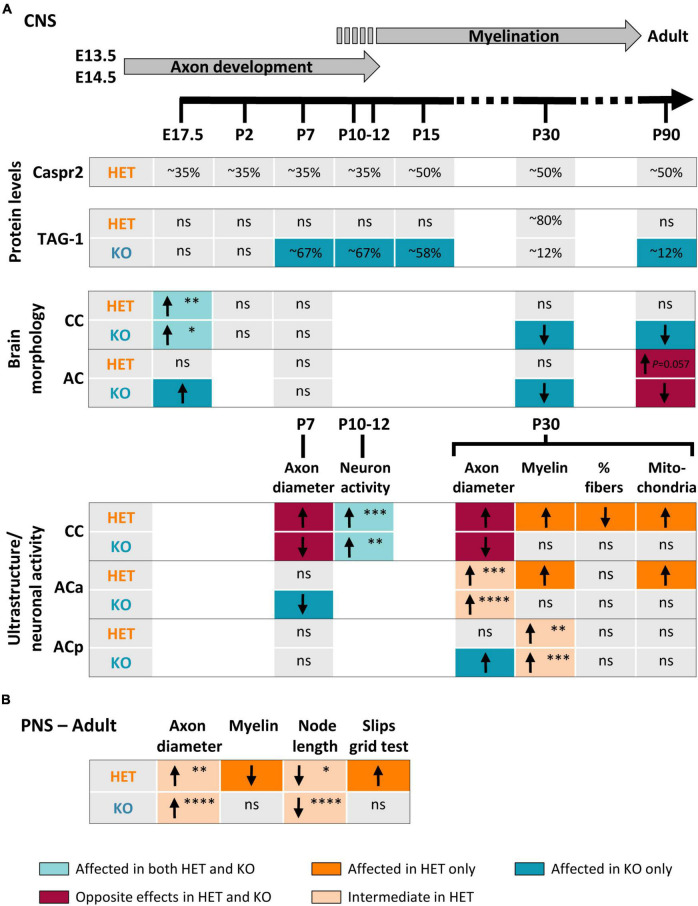
Summary of changes observed in HET and KO mice as compared to wild-type (WT) mice. Changes observed in the brain during development and in adulthood **(A)**. Upper part, timeline of axonal development and myelination in mouse. Changes observed in the sciatic nerves and in the grid test performance **(B)**. Arrows indicate increase (↑) or decrease (↓) in the parameters; ns, non-significant changes. The colors of the boxes underline the nature of the differences between HET and KO (color code at the bottom of the figure). The *P*-values are indicated when HET mice present an intermediate phenotype or tend to be more affected than KO mice (*P < 0.05, ^**^P < 0.01, ^***^P < 0.001, ^****^P < 0.0001). Corpus callosum (CC) E17.5, *P*-values for coronal sections; posterior branch (ACp) myelin P30, *P*-values for G-ratios.

A large variety of homozygous and heterozygous genetic alterations of the *CNTNAP2* gene have been identified in several neuronal disorders (Rodenas-Cuadrado et al., 2014; Hoyer et al., 2015; Poot, 2015, 2017; Saint-Martin et al., 2018). However, the impact of these mutations on Caspr2 functions remains unknown. To better elucidate this issue, we questioned whether *Cntnap2* heterozygosity and *Cntnap2* null homozygosity could impact, either similarly or differentially, some specific functions of Caspr2 during development and in adulthood. Our study indicates that Caspr2 levels influence the gross morphology of both CC and AC, as well as axon diameter at early developmental stages, cortical neuronal activity at the onset of myelination, and axon diameter, myelin thickness and nodal length at later developmental stages (Figure 10). Our observations also highlight Caspr2 functions in axon development and myelination, through which the protein could contribute to normal neuronal network connectivity.

Two previous studies proposed that some functions of Caspr2 in the CNS could be modulated by the level of the protein (Canali et al., 2018; Vogt et al., 2018). Our study reinforces this assumption and further shows that *Cntnap2* heterozygosity and *Cntnap2* null homozygosity lead to complex phenotypes in myelinated fibers. Among all the parameters that we quantified, some present an intermediate phenotype in HET mice as compared to WT and KO mice, some show an opposite phenotype in HET and KO mice, some others are affected in HET or KO mice only, while few tend to be more severely affected in HET mice than in KO mice (Figure 10). The diversity of the phenotypes does not only illustrate differences between HET and KO mice, but also demonstrates that *Cntnap2* heterozygosity and *Cntnap2* null homozygosity impact different types of myelinated fibers differentially. This diversity may be due to the complexity of the mechanisms involved in the bi-directional axo-glial signaling regulating axon and myelin dimensions (which might be disparately affected by the level of protein), and to differences in the molecular mechanisms in which Caspr2 could be implicated from one axon to another. Our observations suggest that these mechanisms could involve the protein TAG-1, which is expressed in both CC and AC axons during development (Kastriti et al., 2019). Caspr2 could also very likely be associated with one or more additional partners among the numerous membranous proteins differentially expressed in CC and AC axons.

According to our observations, Caspr2 could intervene at several time points during the myelination process in the CNS. A handful of elements points to potential roles at the onset of myelination. This includes the switch in morphologies of the CC and AC observed at P7 in mutant pups. Also, as aforementioned, myelination in the CNS relies on a complex sequence of cellular events, which is controlled by OLs intrinsic factors but is also modulated by extrinsic factors, including neuronal activity (Stassart et al., 2018; de Faria et al., 2019). We observed increased neuronal activities in layer III cortical neurons in mutant pups at P10–P12, supporting the possibility that Caspr2 might participate in the cross-talk between the axons of the CC and the OLs at these stages, and possibly later in myelin sheath growth. Caspr2 is involved in the formation and stabilization of neuronal synapses, and in the trafficking of the GluA1 subunits of the AMPA receptors (Gdalyahu et al., 2015; Varea et al., 2015). Of interest, myelination is thought to be modulated by the establishment of axon-oligodendroglial cell synapses and synaptic-like interactions, which respond to the electrical activity of those axons (Bergles and Richardson, 2015). The OPCs display expression of ionotropic neurotransmitter receptors and receive direct glutamatergic and GABAergic synaptic inputs from unmyelinated or partially myelinated axons. In addition, myelin sheath growth involves the establishment of specialized synaptic-like micro-domains at the axon-myelin interface and is regulated by the activity-dependent vesicular release of neurotransmitters along axons (Mensch et al., 2015; Hughes and Appel, 2019). Thus it is possible that Caspr2 plays functions at axon/OLs connections, and possibly also in OL differentiation since single-cell RNA sequencing experiments indicate that Caspr2 is expressed in OLs at different differentiation stages (Marques et al., 2016).

The wrapping of OLs around the axons and the extension of myelin along its length are intrinsically regulated, but modulated by the diameter of the axon they wrap (Stassart et al., 2018). Our data showing axon diameter modifications in the CC and the ACa at P7 indicate that Caspr2 likely contributes to the regulation of the axon diameters of unmyelinated axons before and at the onset of myelination. When considering a single axon, its diameter depends on the axonal cytoskeleton, which is made up of neurofilaments, microtubules, deep dynamic actin trails, and a submembranous actin-spectrin network–which is composed of actin rings regularly spaced by spectrin tetramers (Costa and Sousa, 2021). This membrane periodic skeleton (MPS) can organize transmembrane cell adhesion molecules along neurites and has been shown to regulate multiple aspects of neuronal physiology, including axon diameter, axon-axon interactions, axonal transport, and microtubule stability (Zhou et al., 2022). We previously showed that Caspr2 presents the capacity to interact with actin/spectrin-associated proteins of the 4.1 family (Denisenko-Nehrbass et al., 2003). So, it is tempting to speculate that Caspr2 could participate in the regulation of axon diameter by interacting with the MPS through a 4.1 protein, although it cannot be excluded that Caspr2 could also regulate neurofilament and/or microtubule organization.

Morphological modifications at the ultrastructural levels undoubtedly contribute to the alterations of the gross morphology of the CC and the ACa observed in HET and KO mice during development and at adulthood, but no strict correlation can be established. This is probably due to the fact that, at late development stages, myelinated axons represent only a small percentage of all the axons composing the tracts. In addition, at all developmental stages, the morphology of the tracts may not be dependent on axon diameters only, but also on the compaction of unmyelinated axons. A close observation of the electron micrographs of the CC and AC at P7 seems to show that the organization of the contacts between the axons could be different between WT, HET, and KO conditions (see Figure 6A, compare for example ACa WT and KO), suggesting that Caspr2 could also participate in the regulation of axon-axon contacts. Dedicated image analyses and *in vitro* cellular approaches will have to be developed to investigate this hypothesis. We didn’t analyze the ultrastructure of the CC and AC at E17.5 because their morphology was not sufficiently well-defined to ensure confident measurements. However, it is very likely that the enlargement of the tracts at this stage is also due to an increase in axon diameter and/or modifications in axon-axon contacts, possibly combined with perturbations in the mechanisms guiding growing axons across the midline at early embryonic stages (Nishikimi et al., 2013; Suarez et al., 2014; Fenlon et al., 2021).

The changes in the morphology of the CC and ACa in HET and KO mice during development are interesting with regard to the neurodevelopmental disorders in which *CNTNAP2* alterations have been identified, notably ASD. This disorder is characterized by atypical brain connectivity associated with neural alterations in white matter production and myelination in diverse brain regions (Galvez-Contreras et al., 2020). Numerous structural MRI studies indicate that transient CC overgrowth could be among the earliest neural signatures of ASD in young toddlers (Wolff et al., 2015; Fingher et al., 2017), while disproportionally small CCs relative to overall brain size are among the most replicated imaging findings in older patients (Frazier and Hardan, 2009). We intriguingly observed a similar trend, with an increased thickness in HET and KO embryos at E17.5 and a decreased thickness in KO juvenile and adult mice. Beyond that, our observations support the possibility that the *CNTNAP2* alterations could contribute broadly to myelin defects in ASD patients. Of interest, performing an integrative multi-omics analysis, Jang et al. (2022) recently identified *Cntnap2*-associated ASD networks and found a downregulation in the prefrontal cortex of KO mice of proteins implicated in the formation of the myelin sheath, axon growth, and axonal transport. These data are in line with our current observations and support the role of Caspr2 in axon growth that we described previously (Canali et al., 2018). In addition, they strongly suggest that the increased percentages of myelinated axons containing mitochondria that we observed in the CC and ACa of HET mice could reflect defects in axonal transport.

Finally, we showed that the level of Caspr2 also modulates the organization of peripheral myelinated fibers in adult mice. *Cntnap2* heterozygosity and *Cntnap2* null homozygosity impact the diameter of the axons, the G-ratio, and the length of the nodes differentially. The increase in axon diameters that we observed, notably in KO mice, was not previously reported (Poliak et al., 1999), either because only the diameters of the large-caliber fibers are increased, or because the phenotype was masked in previous morphometric analyses performed on fibers of wide range calibers. The decrease in the nodal length was not previously detected either, although a decreased tendency in the nodal length-to-axonal diameter ratio was reported in KO mice as compared to WT mice (Gordon et al., 2014). When calculating this ratio with our data, it turns out that the decrease measured in KO mice is significant (WT 0.596 ± 0.008, KO 0.510 ± 0.008, Mann–Whitney test *P* < 0.0001), probably because we analyzed a larger number of nodes. Similar to our observations in central myelinated fibers, these abnormalities reveal that Caspr2 plays previously unidentified functions in the development of peripheral myelinated fibers as well. These functions might be similar but also distinct from those in the CNS, since there are substantial differences between the PNS and CNS in the intrinsic and extrinsic signals that dictate axonal ensheathment, myelin thickness, and the formation of the nodes of Ranvier (Nave and Werner, 2014; Salzer, 2015; Rasband and Peles, 2021). Of great interest, we observed that the perturbations of the peripheral functions of Caspr2 have functional consequences in HET mice which present motor and coordination deficits, suggesting that such perturbations in humans could lead to the development of peripheral neuropathies, especially non-demyelinating Charcot–Marie–Tooth disease type 2.

## Conclusion

In conclusion, our study demonstrates that both *Cntnap2* heterozygosity and *Cntnap2* null homozygosity impact axon and myelinated fiber development both in the CNS and the PNS, but in a differential manner. It is a first step indicating that *CNTNAP2* alterations may lead to multiple phenotypes. Further experiments will have to be developed to evaluate the consequences on functional connectivity, especially for HET mice. Another challenge will be understanding how a decrease in Caspr2 level in HET mice on one side, and a total absence of the protein in KO mice on the other side, can eventually lead to different or opposite effects. This will require the characterization of the molecular and cellular mechanisms in which Caspr2 is implicated during axon development and myelination. The switch in the level of Caspr2 in whole brain extracts of HET mice from ∼35% at early developmental stages to ∼50% at later development stages, when compared to WT, supports the possibility that Caspr2 plays major functions before and at the onset of myelination. However, it is unlikely that this switch could be attributed to the functions of Caspr2 in axon and myelin development only. On the contrary, it strongly suggests that *Cntnap2* heterozygosity probably impacts the developmental functions of the protein more broadly. Active phases of dendritic spine formation and synapse development occur from the second week of post-natal development (Farhy-Tselnicker and Allen, 2018). Therefore, our study raises the need to evaluate the impact of *Cntnap2* heterozygosity on the functions of Caspr2 in these processes as well, to further assess the possible consequences of *CNTNAP2* alterations in neurodevelopmental disorders. It would also be of great interest to conduct similar studies for other members of the Contactin Associated Protein family, which have also been associated with neurodevelopmental disorders such as ASD, especially Caspr3/*CNTNAP3* (Vaags et al., 2012; Turner et al., 2017), Caspr4/*CNTNAP4* (Wang et al., 2010; O’Roak et al., 2012; Costa et al., 2022), and Caspr5/*CNTNAP5* (Pagnamenta et al., 2010; Aleo et al., 2020; Ludington et al., 2020). Little is known about the functions of these three proteins compared to Caspr2. However, Caspr3 and Caspr4 have been implicated in synapse formation and/or transmission. Very interestingly Caspr4 was shown to act in a gene dose-dependent manner in the structural maturation of interneuron synapses (Karayannis et al., 2014; Shangguan et al., 2018; Tong et al., 2019), suggesting that the modulation of the biological functions by protein levels could be a common feature to the Contactin Associated Proteins.

## Data availability statement

The original contributions presented in this study are included in the article/Supplementary material, further inquiries can be directed to the corresponding author.

## Ethics statement

This animal study was reviewed and approved by French Ministry of Higher Education, Research and Innovation (Institute agreement D750522; project agreements APAFlS#5496-20160519l5l48075 v8 and APAFIS#31520-2021051709364077 v3).

## Author contributions

CC-D was responsible for the preparation of the samples for electron microscopy and for image acquisition, before performing measurements with GC and MS. GC, CG, and LG performed the brain sections and immunostainings to characterize brain morphology. GC and LG were responsible for the conception or design of the work and carried out the measurements. MG and LG performed the other experiments: sciatic nerve teasing and immunostaining and image acquisition and quantification. GC performed Ctip2^+^ and Satb2^+^ immunostainings and image acquisition and quantification. MD performed electrophysiological recordings. TM performed RT-qPCR experiments. LG performed immunoblots on brain and sciatic extracts and grid-walking test and drafted the work. GC, MG, MD, CL, and LG analyzed the data. All the authors approved the final version of the manuscript.

## Supplementary material

Supplementary Figure 1 Density of callosal projecting neurons in the somatosensory cortex and cortex thickness of E17.5 embryos. Representative images (stacks of 10 confocal images) of the somatosensory cortex (coronal sections) of wild-type (WT), HET, and KO E17.5 embryos immunostained with antibodies directed against Satb2 (green), Ctip2 (red), and L1CAM (white) **(A)**; IZ, intermediate zone; SPL, subplate layer; CP, cortical plate; MZ, marginal zone. Density of Satb2-positive **(B)** and Ctip2-positive **(C)** cells in the somatosensory cortex. Cortex thickness measured at the same levels as corpus callosum (CC) thickness (Figure 2G, levels 1 to 5) **(D)**. **(B,C)** Four animals/genotype, average of measurements on both hemispheres/animal, 25 confocal 1 μm-stacks/hemisphere; **(D)** Six animals/genotype, average of measurements on both hemispheres on one section/animal. Statistical tests: **(B,C)** One-way ANOVA; **(D)** Two-way RM ANOVA.

Supplementary Figure 2 Myelin protein levels in brain extracts. Representative immunoblots showing MBP, MAG, PLP, and GAPDH levels in brain extracts of wild-type (WT), HET, and KO mice at P30 and P90 **(A)**. Levels of MBP normalized to GAPDH levels and relative to mean WT (in %) in brain extracts of mice at P10, P15, P30, and P90 **(B)**. Levels of PLP and MAG normalized to GAPDH levels and relative to mean WT (in %) in brain extracts of mice at P30 and P90 **(C)**. Representative immunoblots showing MBP, MAG, and GAPDH levels in extracts from the neocortex and the corpus callosum (CC) of WT, HET, and KO mice at P30 **(D)**. Levels of MBP and MAG normalized to GAPDH levels and relative to mean WT (in %) in extracts from the neocortex and the CC of mice at P30 **(E)**. **(A,D)** Molecular mass markers positions are shown in kDa on the left of the panels. **(B,C,E)** 6 animals/genotype/age. Statistical tests: Unpaired *t*-test (**E**, CC-P30 MAG, Neocortex-P30 MBP and MAG) or Mann–Whitney test (**B**,**E**, CC-P30 MBP) to compare HET or KO mice to WT mice, and one-way ANOVA test (**B**, P10, P30 and P90; **E**, CC-P30 MAG, Neocortex-P30 MBP and MAG) or Kruskal–Wallis test (**A**, P15; **B**,**E**, CC-P30 MBP) to compare the three genotypes; **P* < 0.05, ^**^*P* < 0.01.

Supplementary Table 1 Statistical tests and results (*P*-values for genotype effect).
