# Supplementary figures and images for: Differential impacts of Cntnap2 heterozygosity and Cntnap2 null homozygosity on axon and myelinated fiber development in mouse
Source: Front Neurosci. 2023 Jan 30;17:1100121. doi: 10.3389/fnins.2023.1100121 (PMC9922869; doi:10.3389/fnins.2023.1100121)

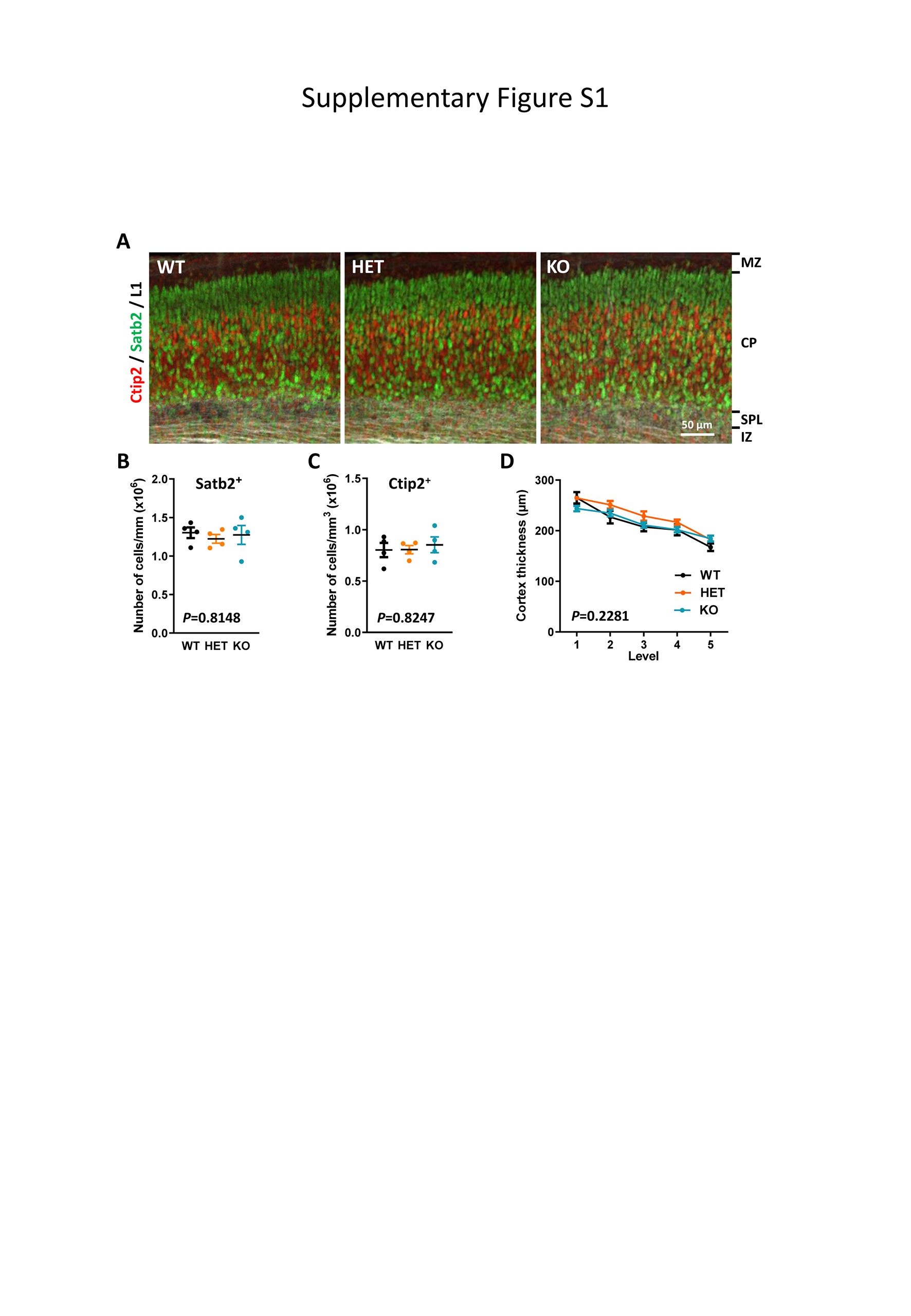

Supplement: Supplementary Figure 1 — Density of callosal projecting neurons in the somatosensory cortex and cortex thickness of E17.5 embryos. Representative images (stacks of 10 confocal images) of the somatosensory cortex (coronal sections) of wild-type (WT), HET, and KO E17.5 embryos immunostained with antibodies directed against Satb2 (green), Ctip2 (red), and L1CAM (white) (A); IZ, intermediate zone; SPL, subplate layer; CP, cortical plate; MZ, marginal zone. Density of Satb2-positive (B) and Ctip2-positive (C) cells in the somatosensory cortex. Cortex thickness measured at the same levels as corpus callosum (CC) thickness (Figure 2G, levels 1 to 5) (D). (B,C) Four animals/genotype, average of measurements on both hemispheres/animal, 25 confocal 1 μm-stacks/hemisphere; (D) Six animals/genotype, average of measurements on both hemispheres on one section/animal. Statistical tests: (B,C) One-way ANOVA; (D) Two-way RM ANOVA. [file Image_1.TIF]

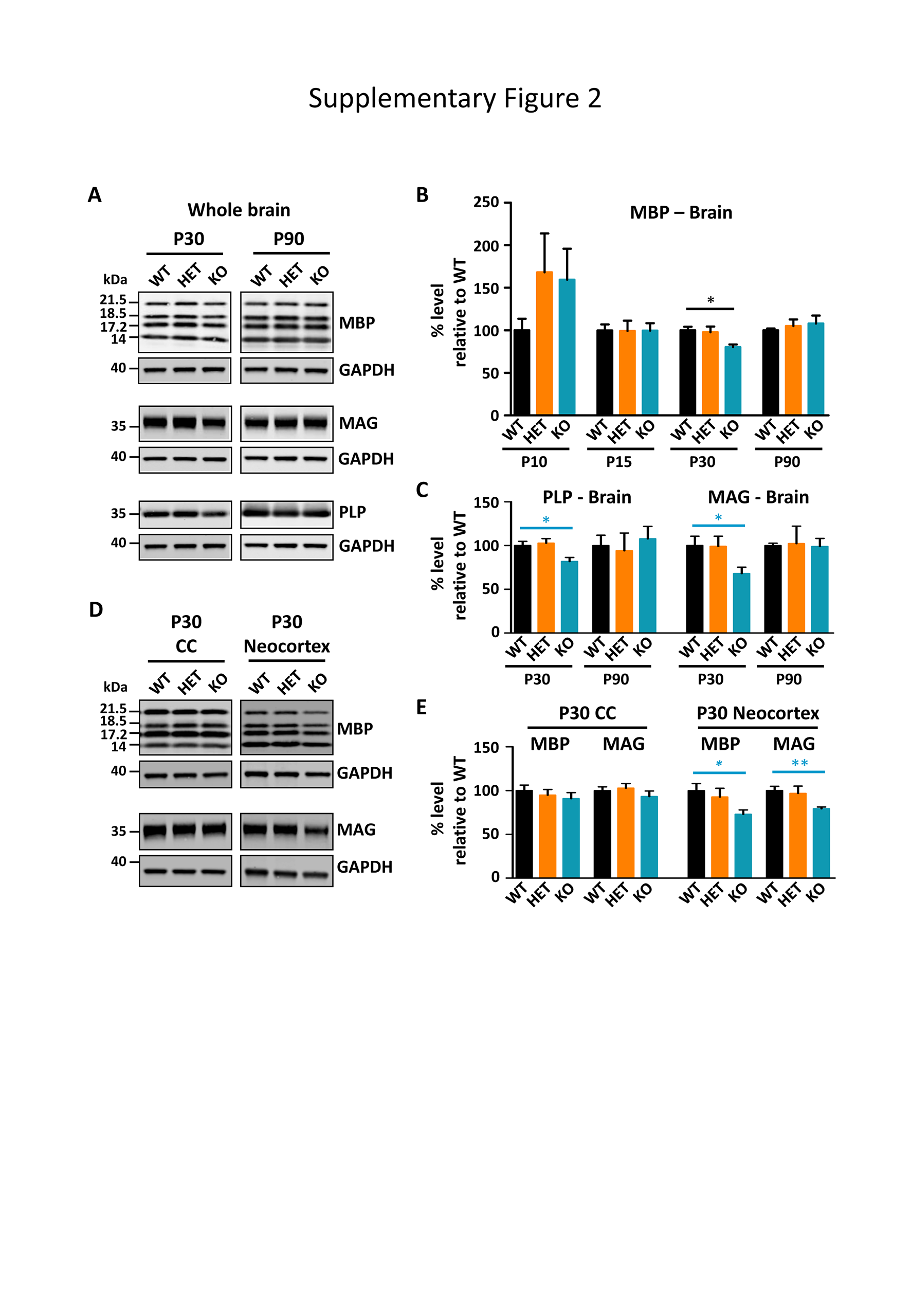

Supplement: Supplementary Figure 2 — Myelin protein levels in brain extracts. Representative immunoblots showing MBP, MAG, PLP, and GAPDH levels in brain extracts of wild-type (WT), HET, and KO mice at P30 and P90 (A). Levels of MBP normalized to GAPDH levels and relative to mean WT (in %) in brain extracts of mice at P10, P15, P30, and P90 (B). Levels of PLP and MAG normalized to GAPDH levels and relative to mean WT (in %) in brain extracts of mice at P30 and P90 (C). Representative immunoblots showing MBP, MAG, and GAPDH levels in extracts from the neocortex and the corpus callosum (CC) of WT, HET, and KO mice at P30 (D). Levels of MBP and MAG normalized to GAPDH levels and relative to mean WT (in %) in extracts from the neocortex and the CC of mice at P30 (E). (A,D) Molecular mass markers positions are shown in kDa on the left of the panels. (B,C,E) 6 animals/genotype/age. Statistical tests: Unpaired t-test (E, CC-P30 MAG, Neocortex-P30 MBP and MAG) or Mann–Whitney test (B,E, CC-P30 MBP) to compare HET or KO mice to WT mice, and one-way ANOVA test (B, P10, P30 and P90; E, CC-P30 MAG, Neocortex-P30 MBP and MAG) or Kruskal–Wallis test (A, P15; B,E, CC-P30 MBP) to compare the three genotypes; *P < 0.05, **P < 0.01. [file Image_2.TIF]
