# Supplementary material for: Differential impacts of Cntnap2 heterozygosity and Cntnap2 null homozygosity on axon and myelinated fiber development in mouse
Source: Front Neurosci. 2023 Jan 30;17:1100121. doi: 10.3389/fnins.2023.1100121 (PMC9922869; doi:10.3389/fnins.2023.1100121)
Supplement: Supplementary Table 1 — Statistical tests and results (P-values for genotype effect). [file Data_Sheet_1.PDF]

					3 genotype comparison						2 genotype comparison				Age comparison for HET mice		
**Figure**	**Structure**	**Age**	**Samples**	**Variable**	**Test**	***P* genotype**	**Post-test**	***P* post-test HET *vs* WT**	***P* post-test KO *vs* WT**	***P* post-test KO *vs* HET**	**Test**	***P* HET *vs* WT**	***P* KO *vs* WT**	***P* KO *vs* HET**	**Test *vs* P90**	**Test *vs* P30**	***P***
**Figure 1B.C**	CC	P90	Coronal sections	CC thickness at different levels<br>CC thickness at midline	Two-way RM ANOVA<br>One-way ANOVA	0.0636<br>0.0109	x<br>Tukey's	x<br>0.6284	x<br>0.0102	x<br>0.063	Two-way RM ANOVA<br>Unpaired t test	0.2145<br>0.4122	0.0153<br>0.0045				
**Figure 1E-G**	CC	P90	Sagittal sections	Area<br>Minimum caliper<br>Maximum caliper	One-way ANOVA<br>One-way ANOVA<br>One-way ANOVA	0.0002<br>0.0243<br>0.1115	Tukey's<br>Tukey's<br>x	0.7676<br>0.4471<br>x	0.0003<br>0.0195<br>x	0.0012<br>0.1906<br>x	Unpaired t test<br>Unpaired t test<br>Unpaired t test	0.573<br>0.24<br>0.4786	0.0004<br>0.0163<br>0.1458				
**Figure 2A**	CC	P30	Sagittal sections	Area<br>Minimum caliper<br>Maximum caliper	One-way ANOVA<br>One-way ANOVA<br>One-way ANOVA	0.0015<br>0.0407<br>0.007	Tukey's<br>Tukey's<br>Tukey's	0.0822<br>0.64<br>0.994	0.001<br>0.0358<br>0.0157	0.0971<br>0.1849<br>0.0128	Unpaired t test<br>Unpaired t test<br>Unpaired t test	0.0531<br>0.4381<br>0.9184	0.0002<br>0.0059<br>0.0022				
**Figure 2B**	CC	P7	Sagittal sections	Area<br>Minimum caliper<br>Maximum caliper	One-way ANOVA<br>One-way ANOVA<br>One-way ANOVA	0.9582<br>0.9212<br>0.6897	x<br>x<br>x	x<br>x<br>x	x<br>x<br>x	x<br>x<br>x	Unpaired t test<br>Unpaired t test<br>Unpaired t test	0.956<br>0.7405<br>0.4657	0.7816<br>0.9858<br>0.4518				
**Figure 2D,E**	CC	P2	Coronal sections	CC thickness at different levels<br>CC thickness at midline	Two-way RM ANOVA<br>One-way ANOVA	0.2762<br>0.3681	x<br>x	x<br>x	x<br>x	x<br>x	Two-way RM ANOVA<br>Unpaired t test	0.1673<br>0.1797	0.458<br>0.8182				
**Figure 2G,H**	CC	E17.5	Coronal sections	CC thickness at different levels<br>CC thickness at midline	Two-way RM ANOVA<br>Kruskal-Wallis	0.0055<br>0.0076	Tukey's<br>Dunn's	0.0057<br>0.0105	0.0314<br>0.3143	0.6736<br>0.5831	Two-way RM ANOVA<br>Mann Whitney	0.0046<br>0.0022	0.0329<br>0.132				
**Figure 3B,D**	AC	P90	Horizontal sections<br>Sagittal sections	Aca thickness<br>AC area	One-way ANOVA<br>One-way ANOVA	0.0164<br><0.0001	Tukey's<br>Tukey's	0.0643<br>0.1629	0.7779<br>0.002	0.0173<br><0.0001	Unpaired t test<br>Unpaired t test	0.0286<br>0.0571	0.5459<br>0.0051				
**Figure 3E**	AC	P30	Sagittal sections	Area	Kruskal-Wallis	0.0139	Dunn's	<0.9999	0.1197	0.0242	Mann Whitney	0.5887	0.0411				
**Figure 3F**	AC	P7	Sagittal sections	Area	One-way ANOVA	0.91	x	x	x	x	Unpaired t test	0.7355	0.6983				
**Figure 3G**	AC	E17.5	Sagittal sections	Area	One-way ANOVA	0.0087	Tukey's	0.9062	0.0115	0.0219	Unpaired t test	0.3076	0.0187				
**Figure 4B-D,F**	CC	P30	Ultra-thin sections	Axon diameter<br><br>G-ratio<br>% of axons with mitochondria<br>% of myelinated fibers	Kruskal-Wallis<br><br>Kruskal-Wallis<br>One-way ANOVA<br>One-way ANOVA	<0.0001<br><br>0.0114<br>0.0096<br>0.0612	Dunn's<br><br>Dunn's<br>Tukey's<br>x	0.1691<br><br>0.0488<br>0.0298<br>x	<0.0001<br><br>0.0181<br>0.6085<br>x	<0.0001<br><br>>0.9999<br>0.0099<br>x	Mann Whitney<br>Kolmogorov-Smirnov test<br>Mann Whitney<br>Unpaired t test<br>Unpaired t test	0.0487<br>0.1314<br>0.0271<br>0.0429<br>0.0135	<0.0001<br><0.0001<br>0.0065<br>0.183<br>0.3735	<0.0001			
**Figure 4E**	CC	P30	Ultra-thin sections	G-ratio as a function of axon diameter							Linear regression/Slope<br>Linear regression/Elevation	0.5891<br><0.0001	0.9058<br>0.6072	0.6916<br>0.0005			
**Figure 5B-D,F**	Aca	P30	Ultra-thin sections	Axon diameter<br><br>G-ratio<br>% of axons with mitochondria<br>% of myelinated fibers	Kruskal-Wallis<br><br>Kruskal-Wallis<br>One-way ANOVA<br>One-way ANOVA	<0.0001<br><br><0.0001<br>0.0053<br>0.0818	Dunn's<br><br>Dunn's<br>Tukey's<br>x	0.0009<br><br><0.0001<br>0.0043<br>x	<0.0001<br><br>>0.9999<br>0.1144<br>x	0.2313<br><br><0.0001<br>0.0609<br>x	Mann Whitney<br>Kolmogorov-Smirnov test<br>Mann Whitney<br>Unpaired t test<br>Unpaired t test	0.0003<br>0.0004<br><0.0001<br>0.0007<br>0.1218	<0.0001<br><0.0001<br>0.4641<br>0.07<br>0.2456	0.1692			
**Figure 5E**	Aca	P30	Ultra-thin sections	G-ratio as a function of axon diameter							Linear regression/Slope<br>Linear regression/Elevation	0.0024<br>x	0.8909<br>0.0913	0.0003<br>x			
**Figure 5H-J,L**	Acp	P30	Ultra-thin sections	Axon diameter<br><br>G-ratio<br>% of axons with mitochondria<br>% of myelinated fibers	Kruskal-Wallis<br><br>Kruskal-Wallis<br>One-way ANOVA<br>Kruskal-Wallis	<0.0001<br><br><0.0001<br>0.3239<br>0.0857	Dunn's<br><br>Dunn's<br>x<br>x	>0.9999<br><br>0.0519<br>x<br>x	<0.0001<br><br><0.0001<br>x<br>x	<0.0001<br><br><0.0001<br>x<br>x	Mann Whitney<br>Kolmogorov-Smirnov test<br>Mann Whitney<br>Unpaired t test<br>Unpaired t test	0.4592<br>0.8077<br>0.0169<br>0.2852<br>0.4	<0.0001<br><0.0001<br><0.0001<br>0.229<br>0.1	0.0004			
**Figure 5K**	Acp	P30	Ultra-thin sections	G-ratio as a function of axon diameter							Linear regression/Slope<br>Linear regression/Elevation	0.9198<br>0.0047	<0.0001<br>x	<0.0001<br>x			
**Figure 6B,E**	Aca	P7	Ultra-thin sections	Axon diameter	Kruskal-Wallis	0.0023	Dunn's	>0.9999	0.0402	0.0023	Mann Whitney<br>Kolmogorov-Smirnov test	0.3686<br>0.5806	0.0132<br>0.0008	<0.0001			
**Figure 6C,F**	Acp	P7	Ultra-thin sections	Axon diameter	Kruskal-Wallis	0.4803	x	x	x	x	Mann Whitney<br>Kolmogorov-Smirnov test	0.4411<br>0.4553	0.6967<br>0.8021	0.4676			
**Figure 6D,G**	CC	P7	Ultra-thin sections	Axon diameter	Kruskal-Wallis	<0.0001	Dunn's	<0.0001	0.0019	<0.0001	Mann Whitney<br>Kolmogorov-Smirnov test	<0.0001<br><0.0001	<0.01<br>0.0144	<0.0001			

					3 genotype comparison						2 genotype comparison				Age comparison for HET mice		
**Figure**	**Structure**	**Age**	**Samples**	**Variable**	**Test**	***P* genotype**	**Post-test**	***P* post-test HET *vs* WT**	***P* post-test KO *vs* WT**	***P* post-test KO *vs* HET**	**Test**	***P* HET *vs* WT**	***P* KO *vs* WT**	***P* KO *vs* HET**	**Test *vs* P90**	**Test *vs* P30**	***P***
**Figure 7B,C**	Somatosensory cortex	P10-P12	Acute coronal slices	Firing<br>Resting membrane potential	Two-way RM ANOVA<br>One-way ANOVA	0.0001<br>0.8806	Šidák's<br>x	0.0002<br>x	0.0031<br>x	0.8085<br>x	Unpaired t test	0.6819	0.6646				
**Figure 8B**	Brain	E17.5<br>P2<br>P7<br>P10<br>P15<br>P30<br>P90	Protein extracts	Caspr2							Mann Whitney<br>Unpaired t test<br>Mann Whitney<br>Unpaired t test<br>Unpaired t test<br>Unpaired t test<br>Mann Whitney	0.0022<br><0.0001<br>0.0022<br><0.0001<br><0.0001<br><0.0001<br>0.0022					
**Figure 8B**	Brain	E17.5<br>P2<br>P7<br>P10<br>P15<br>P30	Protein extracts	Caspr2 in HET mice											Mann Whitney<br>Mann Whitney<br>Mann Whitney<br>Mann Whitney<br>Mann Whitney<br>Mann Whitney		0.0411<br>0.0087<br>0.0022<br>0.0022<br>0.9372<br>0.3939
**Figure 8C**	Brain	E17.5<br>P7<br>P10<br>P30	mRNAs	*Cntnap2* mRNAs							Unpaired t test<br>Unpaired t test<br>Unpaired t test<br>Unpaired t test	0.0194<br>0.0107<br>0.0065<br>0.0001					
**Figure 8C**	Brain	E17.5<br>P7<br>P10	mRNAs	*Cntnap2* mRNAs in HET mice												Unpaired t test<br>Unpaired t test<br>Unpaired t test	0.6052<br>0.1471<br>0.0564
**Figure 8D**	Brain	E17.5<br>P2<br>P7<br>P10<br>P15<br>P30<br>P90	Protein extracts	TAG-1	Kruskal-Wallis<br>One-way ANOVA<br>One-way ANOVA<br>One-way ANOVA<br>One-way ANOVA<br>One-way ANOVA<br>Kruskal-Wallis	0.7552<br>0.2164<br>0.0008<br>0.0062<br>0.0005<br><0.0001<br><0.0001	x<br>x<br>Tukey's<br>Tukey's<br>Tukey's<br>Tukey's<br>Dunn's	x<br>x<br>0.9664<br>0.0909<br>0.9386<br>0.0122<br>0.5831	x<br>x<br>0.0024<br>0.0048<br>0.001<br><0.0001<br>0.0011	x<br>x<br>0.0015<br>0.3142<br>0.002<br><0.0001<br>0.0694	Mann Whitney<br>Unpaired t test<br>Unpaired t test<br>Unpaired t test<br>Unpaired t test<br>Unpaired t test<br>Mann Whitney	0.6991<br>0.7438<br>0.8029<br>0.0698<br>0.7514<br>0.0215<br>0.0649	0.9372<br>0.0727<br>0.0027<br>0.0003<br>0.0003<br><0.0001<br>0.0022				
**Figure 9B,C**	Sciatic nerve	Adult	Ultra-thin sections	Axon diameter<br>G-ratio	Kruskal-Wallis<br>Kruskal-Wallis	<0.0001<br><0.0001	Dunn's<br>Dunn's	0.0025<br><0.0001	<0.0001<br><0.0001	0.0274<br>>0.9999	Mann Whitney<br>Mann Whitney	0.0013<br><0.0001	<0.0001<br><0.0001				
**Figure 9D**	Sciatic nerve	Adult	Ultra-thin sections	G-ratio as a function of axon diameter							Linear regression/Slope<br>Linear regression/Elevation	0.1755<br><0.0001	0.146<br>0.1443	0.0109<br>x			
**Figure 9F**	Sciatic nerve	Adult	Teased fibers	Node length	Kruskal-Wallis	<0.0001	Dunn's	0.0207	<0.0001	<0.0001	Mann Whitney	0.0019	<0.0001				
**Figure 9G**	Sciatic nerve	Adult	Theased fibers	Node length as a function of axon diameter<br>Node diameter	Kruskal-Wallis	0.5342	x	x	x	x	Linear regression/Slope<br>Linear regression/Elevation<br>Mann Whitney	0.4858<br>0.0052<br>0.2644	0.0315<br>x<br>0.8038	0.2559<br><0.0001			
**Figure 9H**		Adult	Grid-walking test	Grid-walking test	One-way ANOVA	0.0011	Tukey's	0.0009	0.3716	0.0256	Unpaired t test	0.0008	0.1882				
**Figure S1B,C**	Somatosensory cortex	E17.5	Coronal sections	Number of Ctip2$^+$ cells/mm^3<br>Number of Satb2$^+$ cells/mm^3	One-way ANOVA<br>One-way ANOVA	0.8247<br>0.8148	x<br>x	x<br>x	x<br>x	x<br>x	Unpaired t test<br>Unpaired t test	0.9662<br>0.411	0.6395<br>0.8468				
**Figure S1D**	Somatosensory cortex	E17.5	Coronal sections	Cortex thickness	Two-way RM ANOVA	0.2281	x	x	x	x	Two-way RM ANOVA	0.1866	0.889				
**Figure S2B**	Brain	P10<br>P15<br>P30<br>P90	Protein extracts	MBP<br>MBP<br>MBP<br>MBP	One-way ANOVA<br>Kruskal-Wallis<br>One-way ANOVA<br>One-way ANOVA	0.3444<br>0.812<br>0.0248<br>0.7331	x<br>x<br>Tukey's<br>x	x<br>x<br>0.943<br>x	x<br>x<br>0.0323<br>x	x<br>x<br>0.0602<br>x	Unpaired t test<br>Mann Whitney<br>Unpaired t test<br>Unpaired t test	0.1833<br>0.4848<br>0.7804<br>0.5419	0.1581<br>>0.9999<br>0.0042<br>0.4308				
**Figure S2C**	Brain	P30<br>P90<br>P30<br>P90	Protein extracts	PLP<br>PLP<br>MAG<br>MAG	Kruskal-Wallis<br>Kruskal-Wallis<br>Kruskal-Wallis<br>Kruskal-Wallis	0.0327<br>0.4657<br>0.0452<br>0.2761	Dunn's<br>x<br>Dunn's<br>x	>0.9999<br>x<br>>0.9999<br>x	0.1197<br>x<br>0.0916<br>x	0.0602<br>x<br>0.1197<br>x	Mann Whitney<br>Mann Whitney<br>Mann Whitney<br>Mann Whitney	0.8182<br>0.4848<br>0.9372<br>0.0931	0.0411<br>0.8182<br>0.0411<br>0.6991				
**Figure S2E**	CC<br>CC<br>Neocortex<br>Neocortex	P30<br>P30<br>P30<br>P30	Protein extracts	MBP<br>MAG<br>MBP<br>MAG	Kruskal-Wallis<br>One-way ANOVA<br>One-way ANOVA<br>One-way ANOVA	0.6001<br>0.4457<br>0.0753<br>0.0201	x<br>x<br>x<br>Tukey's	x<br>x<br>x<br>0.9188	x<br>x<br>x<br>0.0544	x<br>x<br>x<br>0.1114	Mann Whitney<br>Unpaired t test<br>Unpaired t test<br>Unpaired t test	0.4848<br>0.6837<br>0.581<br>0.7502	0.3939<br>0.3957<br>0.0169<br>0.0029				
